# Influence of color on glare perception revealed when seeing the sun through colored glazing

**DOI:** 10.1038/s41598-025-21737-5

**Published:** 2025-10-14

**Authors:** Sneha Jain, Jan Wienold, Luke Hellwig, Marilyne Andersen

**Affiliations:** 1 Civil and Environmental Engineering, Stanford University, Palo Alto, CA USA; 2 Laboratory of Integrated Performance in Design (LIPID), École Polytechnique Fédérale de Lausanne (EPFL), Lausanne, 1015 Switzerland; 3 Department of Science and Math, Fashion Institute of Technology, New York, USA; 4 School of Architecture, Planning and Landscape, University of Calgary, Calgary, Canada

**Keywords:** Daylight, Discomfort glare, Color, Glazing, User assessment, Optics and photonics, Psychology, Psychology

## Abstract

The influence of color on discomfort glare from daylight remains unknown, despite its known effects in electric lighting. This gap limits the ability to predict and mitigate glare in environments with colored glazing and filtered daylight. To address this, we conducted experiments in a controlled daylit office where 56 participants were exposed to four glare conditions induced by the sun visible behind the colored glazing. The conditions differed only in glazing color (red, blue, green, and neutral) towards the sun while having similar visual transmittance resulting in similar glare metrics across colors. Results revealed a strong influence of color with red glazing leading to the highest reports of glare, closely followed by blue, while green and neutral were perceived as least disturbing. These findings suggest that current glare models using photopic luminosity function as a spectral weighting are not effective enough and from this, we assume the Helmholz-Kohlrausch effect can apply to glare similar to brightness perception. To explore this, we tested three color appearance models and supplementary photometry system as alternatives. While these models aligned better with subjective glare reports, they require modifications for higher luminance conditions and need to be tested for wider range of stimuli.

## Introduction

 Windows facilitate daylight and views of the outdoors which have been shown to enhance comfort, cognitive awareness, stress reduction, mood, sleep patterns, and overall health^1–4^. However, excessive daylight can also result in visual discomfort caused by glare, which is especially prevalent in highly glazed commercial buildings. The visible light transmittance and glazing color of windows are key factors that impact the daylight quality and quantity and therefore also impact visual comfort in buildings. Smart glazing, such as electrochromic (EC) glazing, allows for modulation of transmittance to mitigate glare risks and preserve the view to the outside. Recent research has assessed the impact of EC glazing transmittance on discomfort glare perception and established annual transmittance thresholds^5–7^. However, most of the commercially available EC glazing shows a shift in spectral transmission in their darkened state, causing them to appear blue, which can negatively impact certain visual attributes associated with a space^8^. A few studies have investigated the effect of colored façade on visual quality, preference, and/or acceptance indicating that overall warmer-colored daylit environments were consistently found to be more visually acceptable than cooler-colored environments^9–12^. However, the impact of transmitted daylight through colored glazing on discomfort glare has not yet been investigated.

On the contrary, the relation between discomfort glare and the color of^13^ electric light, more specifically, the spectral power distribution (SPD) of LED headlamps, has been studied many times in recent years. A summary of methods and key findings from past studies on the influence of color of electric light on glare can be found in Supplementary Table S1, including one study on the influence of colored daylight on glare. As seen in Supplementary Table S1, earlier studies by Flannagan et al.^14,15^ conducted with monochromatic lights of blue, green, and red colors at six different peak wavelengths have shown that the participants experienced the highest discomfort under blue lamps followed by red and green lamps. The authors also concluded that the CIE spectral sensitivity function V(l)^16^ was not suitable for characterizing discomfort glare. Subsequent studies on colored LEDs, High-Intensity Discharge lamps, and tungsten halogen lamps reported similar results of perceiving higher discomfort glare under shorter wavelengths, while no significant difference was observed in glare between all other peak wavelengths of red, green, yellow, and white colored light sources^17–24^. Based on these findings, some studies have proposed discomfort glare spectral sensitivity functions that were aimed at replacing the CIE *V*(l) function. These proposed functions, although different from each other, all have a higher weighting in the short wavelength region compared to *V*(l), while the mid- and long-wavelengths have similar weighting as *V*(l)^16^. In addition to SPD, several studies have also evaluated the impact of a light source’s CCT (Correlated Color Temperature) on glare perception and again found that the light sources with higher CCTs (blue appearance) caused higher discomfort glare than those with lower CCTs^25–27^.

All the studies with electric lighting have consistently demonstrated an effect of glare source spectra on glare perception and have repeatedly found that people perceived glare more strongly under blue-colored LEDs compared to all other colored LEDs. Recently, a similar trend was found in a user study conducted with daylight, comparing glare perception under blue EC glazing and color-neutral glazing^6^. The study by Jain et al. showed that the participants experienced discomfort glare more strongly in blue EC glazing compared to the color-neutral glazing. This effect, revealed under daylight, could however not be anticipated by applying any of the discomfort glare spectral sensitivity functions proposed by previous studies done with electric light^6^. This finding, therefore, suggests that there is a need to modify the spectral sensitivity functions in the context of glare from spectrally transformed daylight sources.

Given the increasing use of colored building-integrated photovoltaics (BIPV) glass in building façades, determining discomfort glare risks under colored facades is of practical significance. The growing popularity of BIPV façades, driven by the increasing demand for nearly Zero Energy Buildings, has led to an estimated annual growth rate of 40%^28^. Research suggests that colored PV glass is better suited to increase the adoption of BIPV in glass façades due to its added architectural value^29–31^. The use of new generation photovoltaics, such as organic photovoltaics, dye-sensitized solar cells, luminescent solar concentrators, and perovskites, with their wide range of colors and transparency, has the potential to further increase their application in both new and retrofitted buildings^32,33^. However, the spectral shifts in daylight caused by differently colored BIPV façades can have different impacts on the visual quality and comfort of indoor spaces. Furthermore, colored glass that is highly efficient in terms of power generation, such as blue, can be less efficient in terms of visual comfort requirements as evidenced in past studies^28,34^. Therefore, it is necessary to know how the spectral shifts in daylight caused by colored glass façades may impact discomfort glare perception.

To answer this question, we present a user study, conducted in a daylit office-like test room with colored glazing, to determine the influence of color of the sun disc on discomfort glare perception. Study participants were exposed to four daylight conditions having red, blue, green, and neutral-colored glazing as a filter to the sun disc and were asked to report their glare perception under each condition. To the best of our knowledge, this is the only study that investigates the influence of spectral shifts in the light received directly from the sun disc on discomfort glare.

### Objectives

The objectives of this study are: (1) To determine the influence of the color of a glare source (sun disc’s color altered by colored glazing) on subjective glare perception. (2) To determine whether the effect of color, if any, would sustain at two different glazing transmittance levels.

## Method

This study follows a single-blind psychophysical procedure where we examine the relationship between glare from colored daylight (a physical stimulus) and participants’ glare perception (psychophysiological response).

### Study design

**Fig. 1:**
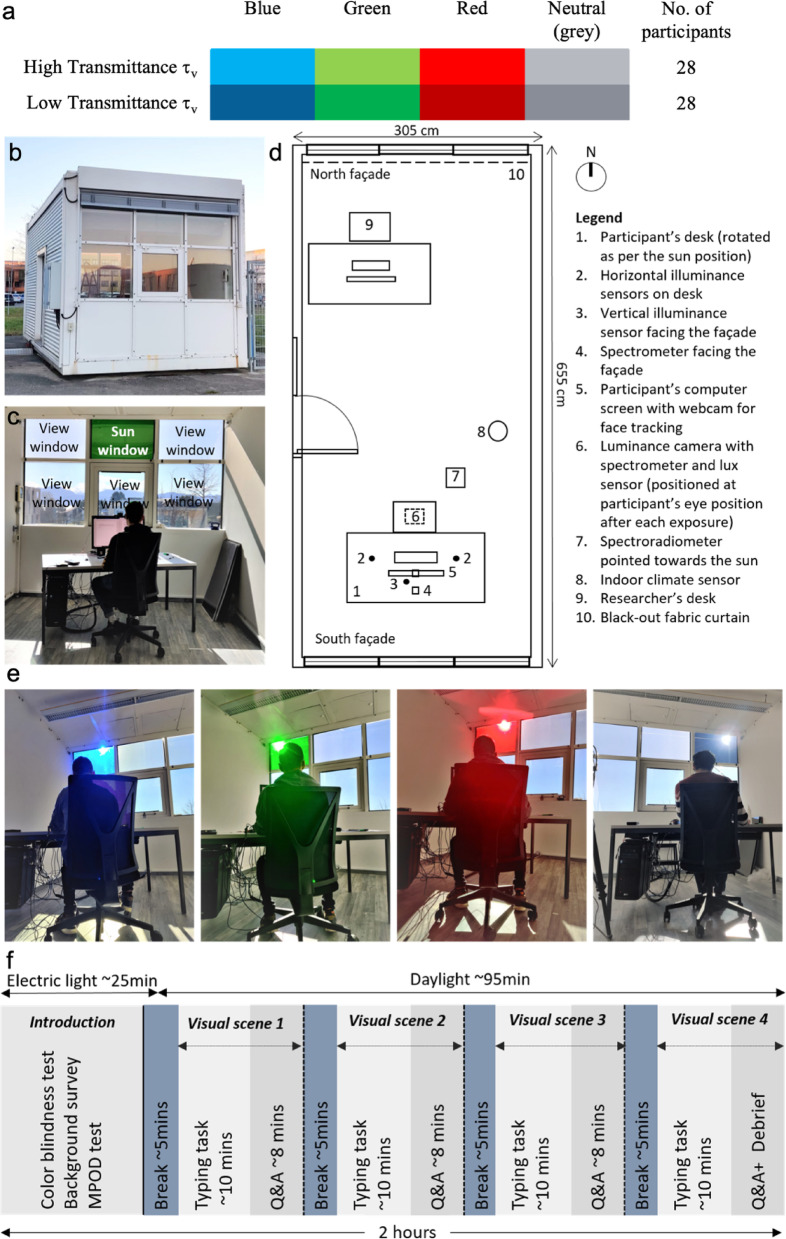
Experiment design, setup and procedure: (**a**) Graphical representation of 2 × 4 full factorial experiment design, (**b**) South façade of test room from outside, (**c**) Test room from inside with the participant (note that sun window can be any of the three upper panes), (**d**) Test room layout with location and description of the equipment, (**e**) Example images of four scenes shown to each participant with glazing colors blue, green, red, and neutral from left to right, (**f**) Experiment procedure.

We designed a 2 × 4 full factorial experiment to determine the influence of colored daylight of similar intensity on participants’ glare perception. A total of eight combinations of experimental scenes were achieved with two levels of glazing transmittances (~ 0.37% and ~ 2.5%) and four levels of glazing colors (neutral, blue, green and red). The color of the glazing varied within subjects while the visible light transmittance of the glazing varied between subjects, resulting in a mixed factorial design as shown in Fig. 1a. This design made it possible to study the influence of colored daylight while keeping the daylight intensity (glare source luminance, background luminance, and vertical illuminance at the eye) similar for a participant. Additionally, a mixed factorial design was selected as it requires fewer participants and offers greater statistical power^35^. As a result, every participant was exposed to four colors of filtered daylight (whose order was counterbalanced across the participants) and only one level of glazing transmittance.

The sample size was derived based on an a priori power analysis in the G*Power 3.9.1.7 tool^36^ with two groups and four repeated measurements, assuming an effect size of 0.30, an alpha value of 0.05 and a power of 0.95. This resulted in a required sample of 50 participants. Considering the possibility of human errors and other technical errors, we recruited a total of 56 participants. Following the mixed factorial design, two groups of 28 participants were exposed to one of the two transmittance levels and the four glazing colors.

### Ethics

The project protocol was approved by the cantonal ethics commission of Canton Vaud, Switzerland Commission- Cantonale d’éthique de la recherche sur l’être humain (CER-VD, ref. No. 2020 − 00667). We confirm that all methods were performed in accordance with the principles of the Declaration of Helsinki. Participants gave written informed consent before the experiments and were compensated as per the local regulations.

### Study participants

A total of 56 individuals (39 male, 17 female) aged between 18 years to 30 years (mean age = 22.6 years) participated in our experiments. The requirements for selection were to be in healthy conditions, not diabetic, have normal color vision (tested using Ishihara and D-15 disc arrangement test^37,38^, have no other visual impairment, have a BMI within the normal ranges, have a non-extreme chronotype (chronotype assessed using Morning-Evening Questionnaires^39^, not to use drugs and not depend on alcohol, to be aged between 18 and 35 years. Since all these parameters can impact participants’ indoor comfort perception, we followed them strictly^40,41^. Other criteria were to have an English proficiency level C1 or higher since the language of instructions and questionnaire was English. To avoid response bias, other inclusion criteria were to not recruit from the disciplines related to the investigated field (i.e., architecture and civil engineering), and to not have any link to the researchers’ topic or the laboratory.

### Test facility and measurements

The study was conducted in an office-like test chamber (Fig. 1b) on the EPFL campus in Lausanne, Switzerland (46°31’00.4” N, 6°33’47.1” E). Figure 1d shows the test room layout and Fig. 1b and c show the images taken from inside and outside the room. Tests were conducted on sunny days with clear skies and stable weather conditions. Daylight through the south façade was the only source of light during the experiments while the north façade was completely blocked by a white curtain. Electric light was used only for the introduction phase of the experiment (cf. Experimental procedure section). Due to the site location and climate, experiments could only be conducted during sunny days between October and March (2021–2022) from 09h00 to 15h00 to get sun exposure and benefit from the low sun angles.

Participants were given a work desk with a computer screen facing the south façade (Fig. 1b). Participant’s desk was rotated as per the sun position to always have the sun visible in their central Field Of View (FOV) as a glare source during the exposure. Another desk with a computer at the back of the room was used by the researcher facilitating the experiments. The room was equipped with instruments to measure the indoor thermal and visual parameters that include room temperature, daylight’s SPD, horizontal and vertical illuminance, the luminance distribution within the participant’s FOV. An indoor climate meter (Testo 480), placed near the participant’s desk (Fig. 1d) continuously measured air temperature, relative humidity, airflow, and CO_2_ content of the test room. The temperature was kept within a comfortable range (21 ± 2 °C). The participant’s desk was equipped with four illuminance sensors (Hagner Special Detector SD2) to continuously measure the light levels. Two of these sensors were installed on the left and the right of the participant’s desk to measure horizontal illuminance. The remaining two sensors were installed at the front and the back of the participant’s computer screen to measure vertical illuminance. We used an absolute-calibrated luminance camera (LMK 98 − 4 Color HighRes by Technoteam equipped with X, Y (V(λ)) and Z filters) to capture luminance and High Dynamic Range (HDR) color images at the participant’s eye level before and after each experiment condition. The camera has a fisheye lens (Dörr Digital Professional DHG, equidistant projection) with a FOV of 160° post-calibration and two Neutral Density (ND) filters of ND1.8 (combined factor 3134.8) to capture the sun without any pixel overflow. We also mounted, at the participants’ eye level, a hand-held illuminance sensor (LMT) to measure the vertical illuminance and a spectrometer (Jaz OceanOptics) with a cosine corrector below the camera lens to measure spectral irradiance. All measurements at the participants’ eye level were done twice for each daylight exposure: at the beginning and at the end of the exposure.

In addition, a spectroradiometer (CS-1000 A by Konica Minolta) was used to measure the SPD of the sun disc visible behind the colored glazing at the participant’s eye level. The CS-1000 A is equipped with an ND3 filter (factor 755.7) and a standard SLR (single lens reflex) optical system that allows precise targeting with a measuring angle of 1°. The viewfinder attached to the spectroradiometer was used to point the sensor at the sun to measure the SPD. All the ND filters (Type: B + W) used in the experiment exhibit a nearly constant spectral transmission which was essential to capture the colored sun without any error. Since many commercial ND filters have high transmittance in long-wavelength regions, therefore, all the filters were measured to ensure constant spectral transmission. We also measured global (total) and diffuse solar irradiance (W/m^2^) with a Pyranometer installed near the test room location (SPN1, wavelength range: 400–2700 nm) to monitor the weather conditions. All the continuous measurements were done at a 30 s interval.

In parallel to environmental measurements, we also recorded participants’ pupil size using eye-tracking glasses, Pupil Core^42^, that participants wore during the exposure to experimental conditions. Additionally, a webcam was used to record their faces during the exposure to extract their gaze behavior and head movement from the recordings using a deep learning-based tool called Openface^43^.

### Experimental conditions

Each participant was exposed, in randomized order, to four experimental conditions differing only in the color (either red, blue, green, or neutral) of one upper windowpane facing the sun (Fig. 1e and labeled ‘sun window’ in Fig. 1c). The position of this colored windowpane was changed during the experiment based on the sun’s position. To preserve natural interior color rendering and avoid bias from altered color perception, only the sun window was modified while the remaining windowpanes (labeled ‘view windows’ in Fig. 1c) were kept at the same visible transmittances of t_v_ =8.28%. This transmittance was chosen to allow reaching the recommended light level on the work desk (at least 500lux^44^ while avoiding glare from the view windows.

**Fig. 2:**
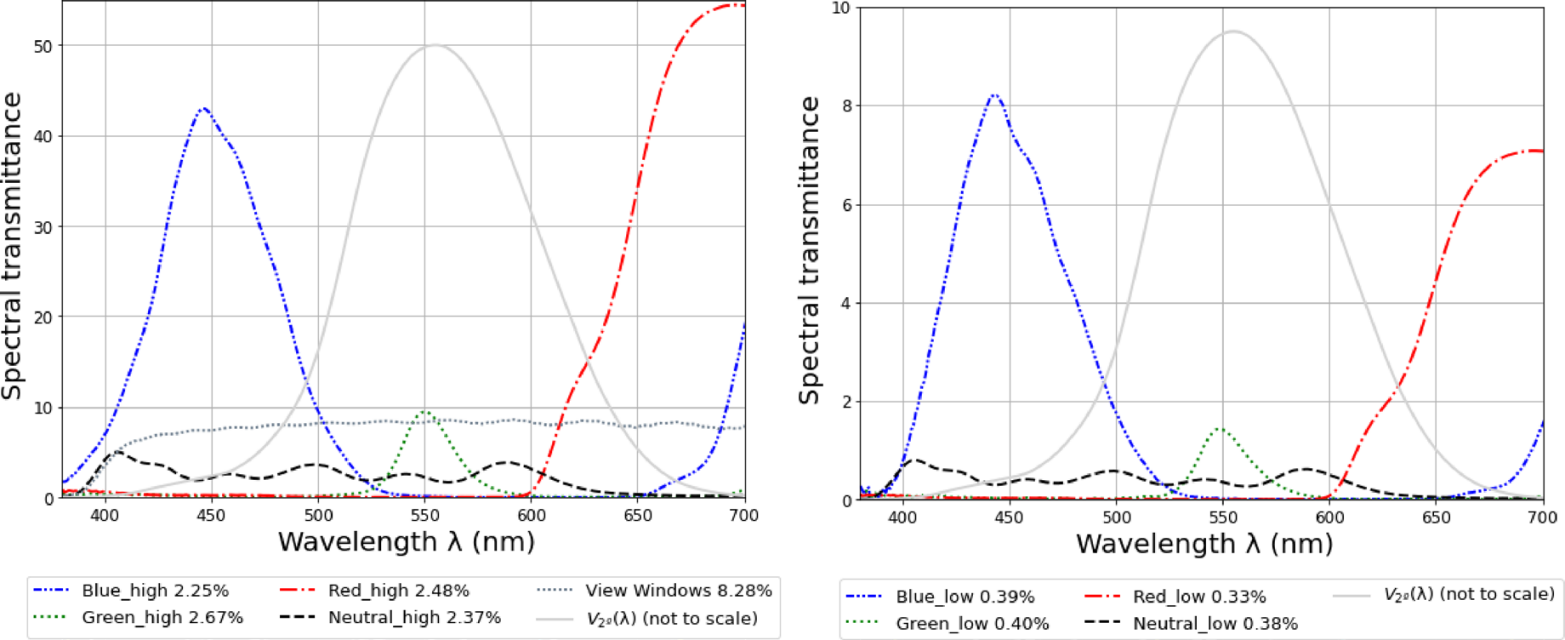
Measured spectral transmittances of colored glazing with high transmittance levels (left) and low transmittance levels (right) in comparison with the CIE 1988 V_2°_(l).

Two levels of visible transmittance (low and high) were used for the sun window across all four colors, resulting in eight experimental conditions: Red_low, Green_low, Blue_low, Neutral_low; and Red_high, Green_high, Blue_high, Neutral_high. These transmittance values (cf. Table 2) were kept similar across the four scenes shown to each participant with average transmittance values for the high and low levels as 2.5% and 0.37%, respectively. These values were selected to create two non-overlapping levels of sun luminance that would elicit varied glare responses based on a past study with neutral glazing where glare perception was evaluated at low levels of transmittances^6^. The glazing colors were selected to minimize spectral overlap and span distinct parts of the visible spectrum as seen in measured spectral transmittance plots (t_v, n−h_) in Fig. 2. The glazing selection process, measurement method, and properties are further detailed in supplementary material under the section *Supplementary Method*, Supplementary Table S2, Supplementary Figures S1 and S2.

### Experimental procedure

Each experimental session lasted about two hours with one participant at a time and a maximum of two participants in a day. The 2-hour-long procedure is visualized in Fig. 1f. The first part (named “Introduction” in Fig. 1f) was conducted under electric light with closed window blinds, during which participants were introduced to the test procedure following a single-blind approach to avoid response bias^45^. Afterward, they answered a background survey. This was followed by a short (~ 10 min), non-invasive measurement of the participant’s *MPOD* (Macular pigment Optical Density) for both eyes as a part of another project related to the influence of MPOD on glare sensitivity^46^.

After the introduction step, participants were exposed to four colored daylit scenes, with dark-adapted breaks in between. Each scene started with a typing task (~ 10 min), followed by answering survey questions (~ 8 min), and ended with a break (~ 5 min). During the break, participants were given an eye mask to cover their eyes and headphones to listen to music while the researcher prepared the test room for the next scene by opening the window blinds, changing the color of the sun window, and rotating the participant’s desk to face the colored gazing and see the sun in their FOV. During that same break, the researcher also captured an HDR image, measured the vertical illuminance, the spectral irradiance at the participant’s eye level, and the spectral irradiance of the sun visible behind the colored glazing. After the break, the participants were asked to type a predefined text shown on the screen for 10 min to simulate an office environment. More importantly, this task allowed them to visually adapt to the lighting condition. The text to be typed varied between visual scenes and all texts had been evaluated beforehand as having the same level of readability (checked with^47^. Afterward, the participants answered a comfort survey reporting their thermal, visual, and color perception. This part took from 6 min to 10 min (average ~ 8 min) depending on each participant’s speed. This sequence was repeated for the four scenes. The whole experiment ended with a debriefing survey where the participants provided their overall feedback on the comfort they perceived in all four conditions.

### Survey questionnaire

Participants answered three survey questionnaires during the experiment:

(i) Background questionnaire (about demographics, current emotional and physical state, and indoor comfort preferences asked at the beginning of the experiment).
(ii) Comfort questionnaire (about thermal comfort, discomfort glare, lighting levels, and color perception asked after every exposure).
(iii) Debriefing questionnaire (about view quality, comparative feedback on four scenes, overall feedback asked at the end of the experiment).

**Table 1:** Questionnaire items asked after every exposure.

Category	Question	Response labels
Open-ended	Q1. Is there anything about the physical environment that disturbs you in this moment?	Text
Thermal comfort	Q2. At this precise moment, how are you feeling?	Cold –Cool –Slightly cool –Neither cool nor warm –Slightly warm –Warm –Hot
	Q3. How satisfied are you with the thermal situation in this room?	Very dissatisfied – Dissatisfied –Neither dissatisfied nor satisfied – Satisfied – Very Satisfied
Visual comfort/ Glare	Q4. Are you experiencing any discomfort due to glare at the moment?	Yes- No
	Q5. At the moment, how would you describe glare in your field of view?	Imperceptible – Noticeable– Disturbing – Intolerable
	Q6. How much discomfort due to glare are you experiencing at the moment?	Not at all – Slightly – Moderately – Very much
	Q7. On a scale of 0–10, how much discomfort due to glare are you experiencing at the moment?	Not at all 0-1-2-3-4-5-6-7-8-9-10 Very much
Color perception	Q8. The colors of object inside the room looks natural	1-Strongly disagree − 2-3-4-5-6-7-Strongly agree

This section details the questions, listed in Table 1, focused on color perception, visual and thermal comfort that are analysed in the current study (cf. Results section). The questions and response labels were adapted from past studies with an aim to minimize the potential response bias^48–50^. The order in which the questions were asked was randomized in the survey to avoid any order bias^51^. These questions were answered on either a binary scale, a Likert (ordinal) scale, or a linear scale, or in one case as a free text, as specified in Table 1.

The first survey question was an open-ended text field (Table 1, Q1) that allowed participants to report any disturbing sensations without forcing them to select from pre-defined options or drawing their attention to a particular comfort parameter^49^. We evaluated answers to this question to check whether participants spontaneously mentioned glare in their answers since this was the main independent variable in the tested conditions. Thermal comfort and color perception (Table 1, Q2, 3, 8) were evaluated only for ensuring that they were not causing any confounding effects on our main variables of interest (i.e. discomfort glare). Discomfort glare was the main independent variable that was evaluated on four different scales (Table 1, Q4- Q7) to check and ensure the internal consistency of answers and the reliability of the questionnaire items.

### Data cleaning and processing

We applied data filtering rules to ensure that all the experimental conditions had stable weather conditions and an unobstructed view of the sun within the participants’ FOV. We discarded data points where the deviation in measured global horizontal irradiance (GHI) was more than 25% ((GHI_max_ -GHI_min_)/GHI_mean_) over the duration of a participant’s exposure to the whole session. We also discarded the data where the sun was hidden by a window frame or by any other object in the participants’ FOV (checked by visual inspection of the HDR images). After cleaning the data, we were left with a total of 205 data points (102 with low transmittance and 103 with high transmittance) by discarding 8.5% of the data from the initial dataset of 224 points (~ 56*4). The final 205 data points consist of 50 points in blue conditions, 52 points in green conditions, 53 points in neutral conditions, and 50 points in red conditions. This distribution fulfilled our criteria of having at least 50 points in each color category.

The images of the scene were captured in “.pf” (picture float) format and were converted to “. hdr” format. HDR images were then processed using the Evalglare tool (version 3.03)^52^ to calculate the glare metrics corresponding to the viewed scenes. We extracted the sun disc luminance (cd/m^2^) from the HDR images by selecting the highest pixel value. The glare metric values were calculated based on the default Evalglare algorithm that considers a threshold of 2000 cd/m^2^ for glare source detection. The measured spectral data were processed by using the color package^53^ of Python 3.9.

### Statistical analysis

We used descriptive statistics to summarize the measured and HDR-derived physical quantities associated with each experimental condition. Descriptive statistics included mean, median, standard deviation, scattered boxplots, stacked bar plots, and line plots. Cronbach’s alpha^54^ was used to check the internal consistency between the participants’ glare responses on different questionnaire items. To ensure the similarity of glare metric values between colored scenes, two sample Kolmogorov–Smirnov (K-S) test was applied to check whether they come from the same distribution. To determine the effect of color on glare perception, we applied the Friedman test, which is a non-parametric test that is appropriate for analysing data with repeated measures or related samples when the dependent variable is measured on an ordinal scale^55^. If the Friedman test yields a significant result, we then proceed to perform post-hoc analyses, using Dunn’s test^56^, to determine the specific differences between the pair of colors. We relied on Cliff’s delta to estimate the effect size for comparing two samples with ordinal data and to measures the magnitude of difference between two colored pairs. We conducted multiple pairwise comparisons between the four colored scenes by applying a Bonferroni correction in which the p-values were multiplied by the number of comparisons. The Bonferroni correction was chosen over other methods because it has a stricter criterion and can be applied without any distributional assumptions of the data^57,58^.

## Results

### Thermal comfort evaluations

To maintain adequate thermal comfort, we kept the indoor temperature within comfortable ranges of 21 ± 2 °C. Past studies have shown that glare perception can be influenced by thermal discomfort^59^ and the color of daylight can influence thermal comfort^11^. Thus, to confirm that the thermal conditions were not creating any unwanted biases, we analyzed the participants’ responses to thermal comfort questions (Q2-3 in Table 1). On the ASHRAE 7-point scale of Cold to Warm, 92% of participants reported either “Neither cool nor warm” (41%) or “Slightly warm” (36%) or “Slightly Cool” (15%) whereas only 8% reported either “Warm” (6%) or “Cool” (2%) and no one rated the two extreme ends “Cold” or “Hot”. On the thermal satisfaction scale, 91% answered either “Satisfied” (61%) or “Neither Satisfied nor Dissatisfied” (30%) whereas only 9% rated “Dissatisfied”. Therefore, a majority of participants considered thermal conditions as comfortable. We also performed an additional analysis reported in results section removing those 9% dissatisfied cases to ensure robustness of the findings. Additionally, there were no significant differences in thermal comfort between the four colored conditions (see Supplementary Figure S3). These results confirm that thermal comfort was maintained in the experiments and there is thus a minimal risk of bias imputable to thermal conditions.

### Spectral measurements and color perception

**Fig. 3:**
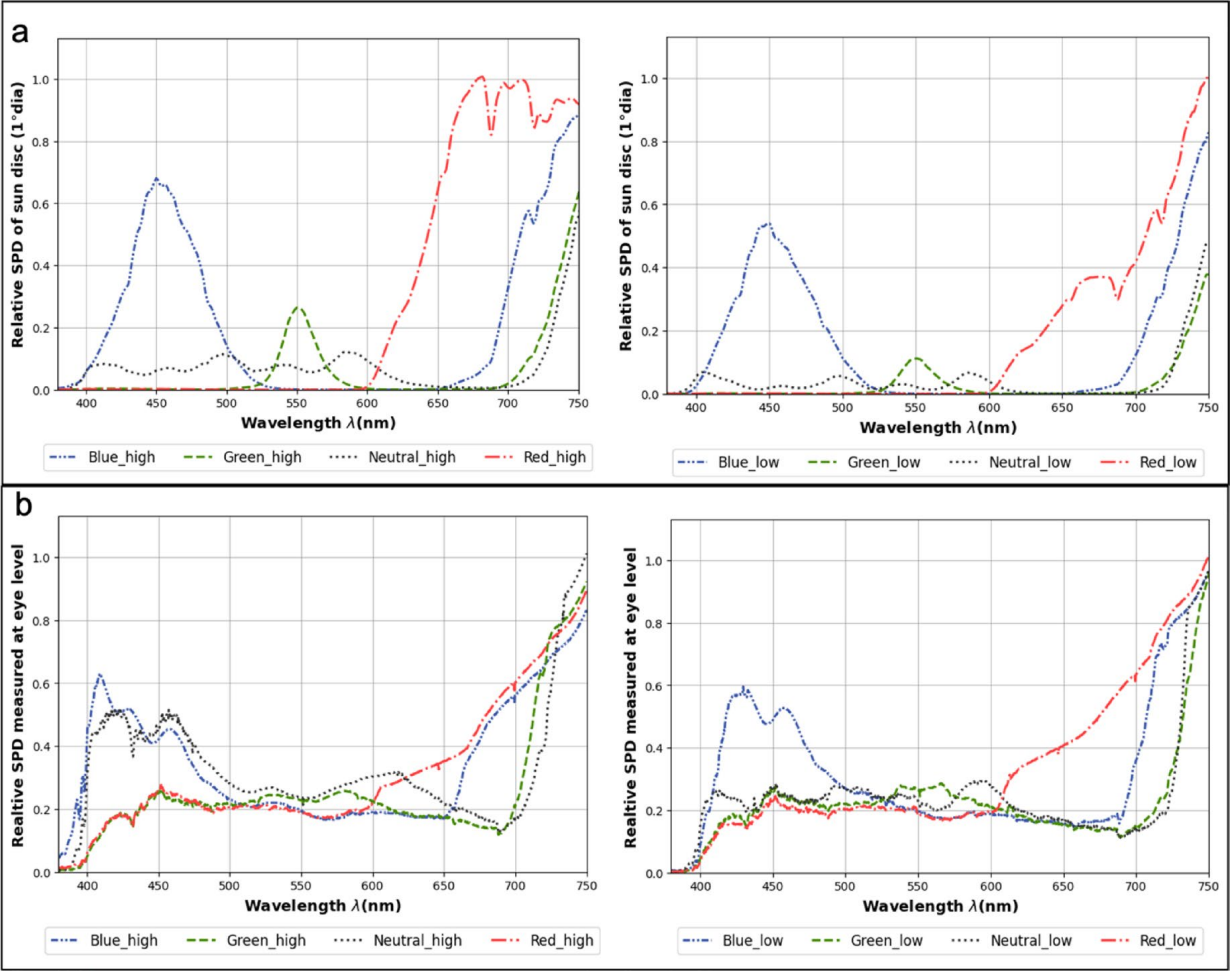
Measured spectral characteristics of the daylight: (**a**) Mean relative SPD of sun disc, (**b**) Mean relative SPD measured at participants’ eye level for all conditions (normalized to 1) with high (left) and low (right) glazing transmittance.

The spectral characteristics of the experiment conditions are described by the measured SPD of the sun disc visible to the participants (Fig. 3a), SPD measured at the participants’ eye level (Fig. 3b) and CCT (Table 2).

The SPD of the sun was measured as an integral of 1° diameter circling the sun disc which is approximately twice the size of the actual sun disc (∅=0.533°). As expected, the SPD of the sun (Fig. 3a) follows the ‘sun window’ spectral transmittance curve shown previously in Fig. 2. It should be noted that the total irradiance under the visible spectrum varies between conditions: while the photometric units were kept similar between conditions, the corresponding radiometric units were, by definition, not. Unlike Fig. 3a, in Fig. 3b, showing the SPD at eye level, the impact of sun disc’s SPD is attenuated due to the contribution of daylight coming through the color-neutral ‘view windows’, which was our strategy to preserve the naturalness of indoor elements by changing only the color of the sun window. The variations in mean measured CCT values (Table 2) between scenes follow the color of the ‘sun window’: the conditions associated with the blue glazing have the highest CCT whereas conditions associated with the red glazing have the lowest CCT. The CCT values are driven more by the sun’s luminance which is almost 10 times higher in high transmittance conditions compared to low transmittance conditions. Therefore, the CCT measurements may not represent the color perception which can be confirmed by analyzing subjective votes on the color perception question (Table 1, Q8). We found that 88% of the participants rated the naturalness of color in the space as 6 or above on the 7-point scale (1-strongly disagree to 7-strongly agree). We can conclude that by maintaining enough color-neutral windows on the façade (all except the ‘sun window’), we were able to avoid any risk of potential bias due to color distortion.

### Discomfort glare evaluations

#### Photometric characteristics of experiment conditions

To isolate the effect of color on glare perception, we ensured that daylight conditions were comparable across the colored scenarios presented to each participant, thereby, making color the only variable within subjects. For this evaluation, we used both measured vertical illuminance at eye level (Ev) and HDR image-derived metrics, including sun luminance, sun position index, viewing angle, and glare metrics- DGP (Daylight Glare Probability), and CGI (CIE Glare Index), as summarized in Table 2. First, we validated the accuracy of the HDR images by comparing the image-derived E_v_ to the measured E_v_ values, resulting in an RSME of 78 lx (7.4% when normalized) and a normalized bias of 7.5%., indicating good-quality images reliable for comparing glare conditions across scenes.

**Table 2:** Summary of the descriptive statistics pertaining to all experimental conditions. The values in round parentheses indicate standard deviation.

Experiment Scene	Blue_ low	Green_ low	Red_ low	Neutral_ low	Blue_high	Green_ high	Red_ high	Neutral_ high
Sample size	25	26	25	26	25	26	25	27
Glazing τv	0.39%	0.40%	0.33%	0.38%	2.25%	2.67%	2.48%	2.37%
Mean CCT (in Kelvin)	17,790	6,350	5,160	6,890	18,926	6,060	5,240	7,970
Mean E_v_ (in lux)	1,115 (109)	1,100 (140)	1,035 (120)	1,090 (127)	2,290 (129)	2,430 (169)	2,350 (133)	2,370 (143)
Mean sun luminance (millions cd/m^2^)	3.43 (0.57)	3.89 (0.50)	2.48 (0.27)	3.41 (0.46)	22.1 (4.26)	26.1 (4.15)	22.3 (3.08)	25.8 (4.42)
Mean DGP	0.38 (0.016)	0.38 (0.02)	0.36 (0.019)	0.38 (0.017)	0.50 (0.02)	0.51 (0.02)	0.50 (0.02)	0.51 (0.02)
Mean CGI	38.7 (1.66)	39.5 (2.09)	37.3 (2.11)	39.5 (1.87)	48.7 (1.76)	49.3 (1.96)	48.9 (1.73)	49.5 (2.09)
Mean position index	2.8 (0.70)	2.8 (0.74)	2.7 (0.76)	2.7 (0.62)	2.8 (0.52)	2.8 (0.51)	2.8 (0.51)	2.8 (0.70)
Mean viewing angle	26.5 (5.32)	26.7 (5.26)	26.2 (5.43)	26.7 (5.36)	26.9 (3.95)	26.8 (4.26)	27.0 (4.38)	26.6 (4.77)

**Fig. 4:**
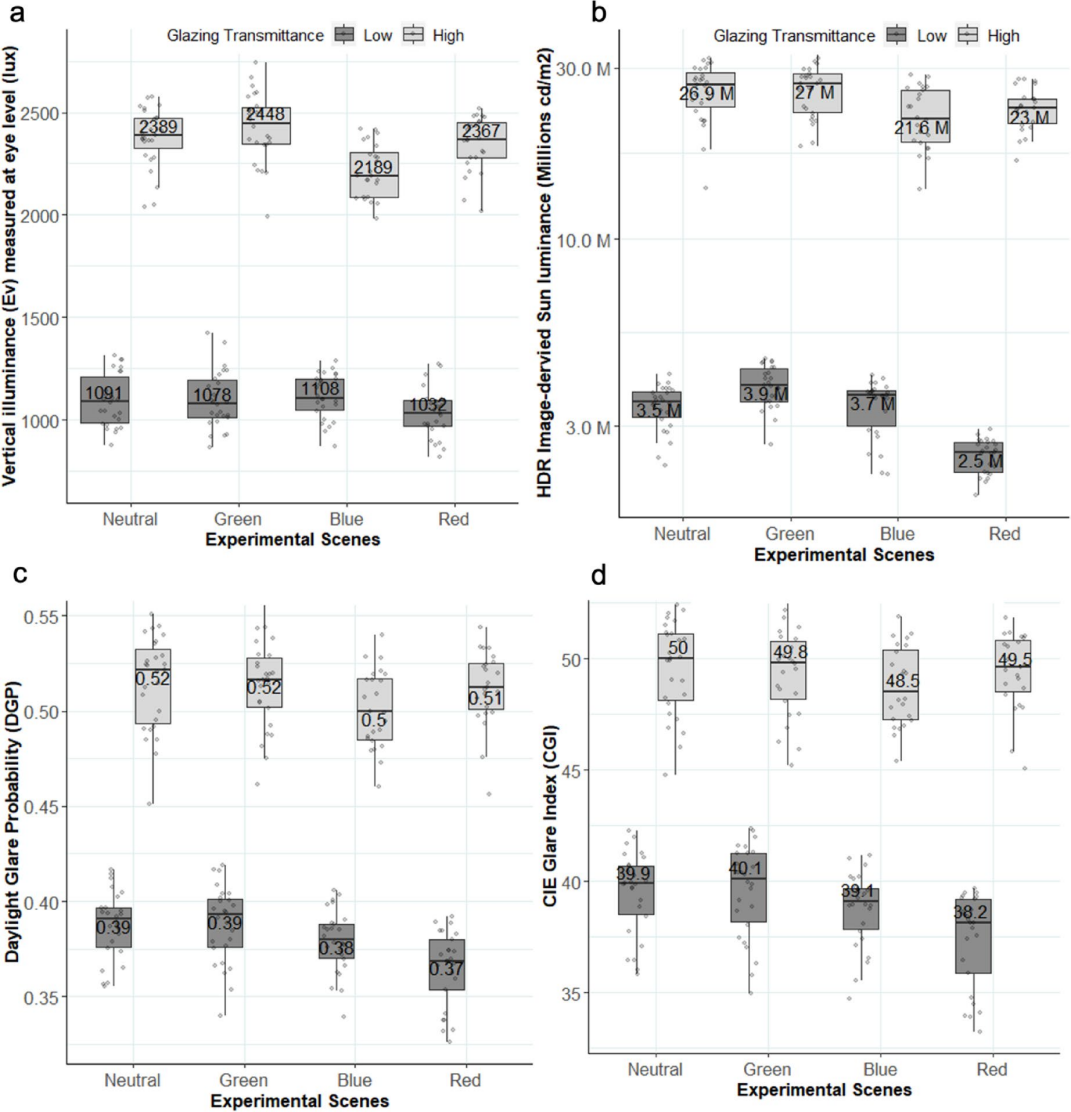
Scattered boxplots of photometric quantities with median values: (**a**) Measured vertical illuminance at eye level, (**b**) HDRI-derived sun luminance, (**c**) Daylight Glare Probability (DGP), (**d**) CIE Glare index (CGI) for eight experimental conditions.

As shown in Table 2; Fig. 4, mean values of the E_v_, sun luminance, glare metrics, position index and the viewing angle to the sun were similar across four colors within each transmittance level. Some variations in E_v_ can be seen as slightly lower levels in the Blue_high and Red_low conditions and slightly higher levels in the Green_high condition compared to all the others (Fig, 4a) owing to the slight differences in their glazing transmittances (Table 2). However, the mean difference in E_v_ between the scenes remains as low as 15.7% generally not enough to be noticeable subjectively since a change by at least 1.5 times is required to create a difference in lighting perception^60,61^. We can observe similar variations in sun luminance and glare metric values (CGI and DGP) in Fig. 4b, c and d. Out of these quantities, the difference observed in the sun luminance for the Red_low condition is largest due to lower glazing transmittance (cf. Table 2). A K-S test showed that glare metrics (DGP and CGI) were similar across the colored scenes except for the low-intensity pairs of neutral-red and green-red scenes. In both pairs, red stimuli had statistically significantly lower glare values (*p* < 0.05) than the neutral and green ones. To assess whether this difference could influence our findings, we referred to the glare thresholds established in a prior cross-validation study^62^ where a change in glare perception by one category on a four-point scale requires a difference of at least 8% in CGI or 11% in DGP. In our study, the maximum observed difference in CGI and DGP between any scene is under 8%, suggesting that the conditions can be considered perceptually comparable. We further examine the implication of these small differences in the next section on subjective responses.

#### Participants’ responses

**Fig. 5:**
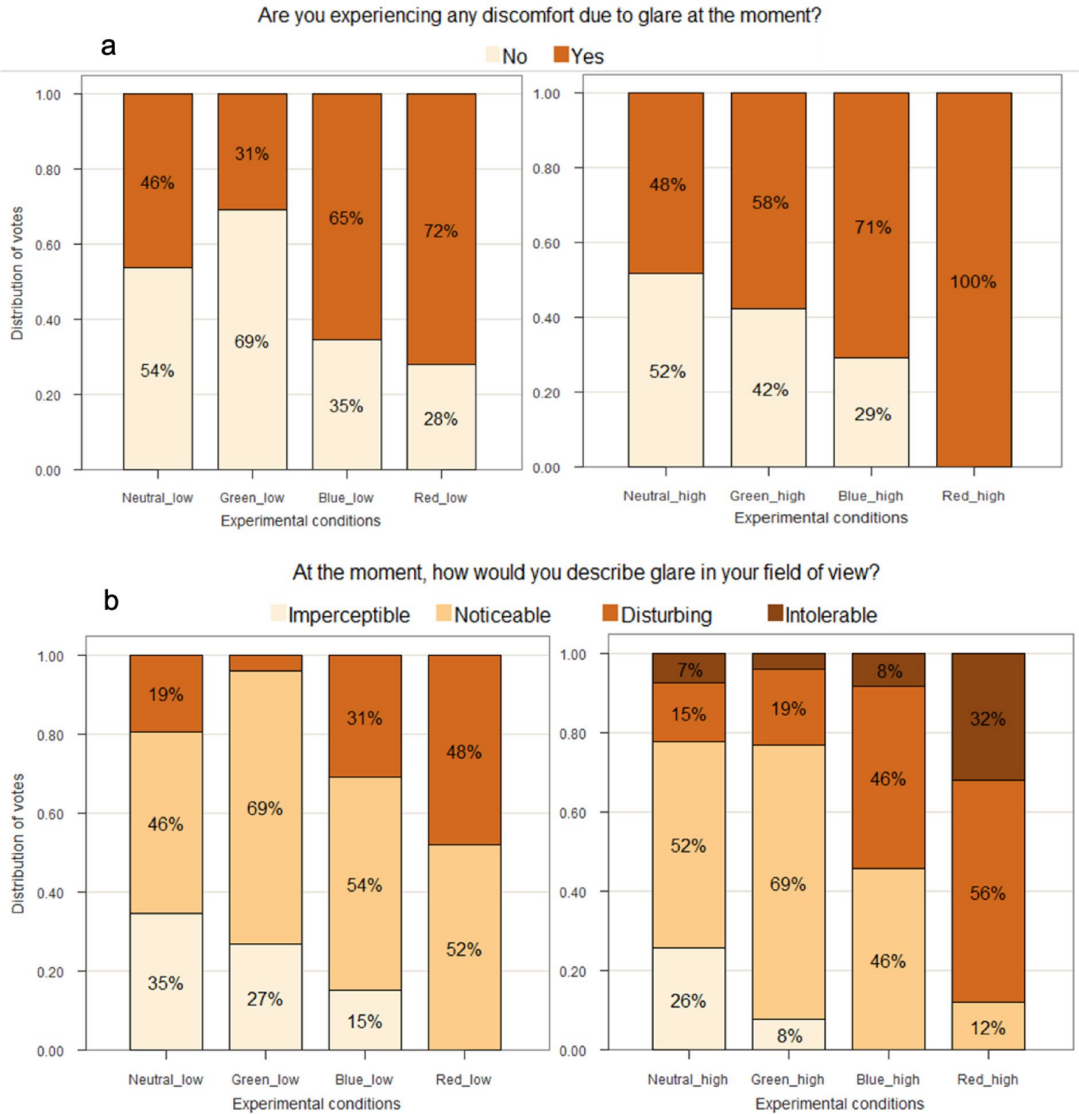
Participants’ responses to discomfort glare on (**a**) binary response labels, and (**b**) four-point ordinal labels for conditions with low (left) and high (right) glazing transmittance.

Figure 5 presents participants’ responses to discomfort glare questions for eight experimental conditions. Analysis of the text-answers from open-ended question (Table 1, Q1) revealed that among those who answered “Yes” to the binary glare question (Q4), 73% spontaneously mentioned terms like glare, sun, or bright/colored light as a disturbing factor, thus confirming that glare was a salient environmental factor. Furthermore, in an additional paper-based question where participants marked the location of the glare source on a perspective view of the scene, 96% of participants identified only the sun location as the source of glare. We found an excellent internal consistency (Cronbach’s alpha = 0.94) between the participants’ answers to the three glare questions (Table 1, Q5-7). Therefore, we focus the analysis on the responses to Q4 (binary) and Q5 (ordinal) commonly used in glare research^63–66^.

In Fig. 5a, we can observe a clear difference in the participants’ glare responses despite similar glare metrics across colored conditions. Participants reported glare more often under red and blue glazing compared to neutral or green, across both low and high transmittances. Under low transmittance (Fig. 5a, left), Green_low was rated most comfortable, while Red_low was least, with 41% more participants reporting glare than for Green_low. Similarly, under high transmittance (Fig. 5a, right), Neutral_high was rated most comfortable, and Red_high least, this time with all participants reporting glare (i.e. 52% more participants than for Neutral_high). The differences are thus much higher between the neutral or green and the blue or red than when comparing green to neutral or blue to red. These differences were unexpected given the similar glare metrics, except for the blue-vs-neutral comparisons which aligns with findings from a recent study^6^ where blue EC glazing was found to create higher discomfort glare compared to neutral glazing.

Overall, high transmittance conditions induced more glare (16% more on average), as expected due to higher source luminance. The pattern of glare responses was largely consistent across transmittance levels, except for the green glazing which was rated most comfortable in the low group but second to neutral in the high group, though this difference was not statistically significant, further discussed in the next section.

Ordinal responses on imperceptible to intolerable scale followed the same trend (Fig. 5b). Participants were overall more disturbed by glare under blue and red glazing compared to green and neutral. Under low transmittance, Red_low was reported most disturbing (48% disturbed), while Green_low was least (4% disturbed). Under high transmittance, again Red_high remained the most disturbing (88% reported disturbing or intolerable glare), followed by Blue_high (63%), while Neutral_high and Green_high were least disturbing (22% and 23%, respectively).

When responses across transmittance levels were aggregated, the proportion of participants reporting disturbing or intolerable glare was highest for red glazing (69%), followed by blue (42%), neutral (22%), and green (14%). This suggests that red glazing was perceived as the least comfortable, and green the most, outperforming even neutral glazing, though differences were not statistically significant, as discussed in the next section. Regarding the blue glazing, the number of participants reporting disturbing glare was approximately three times higher than green and twice as many as neutral. Median glare rating on the 0–10 numerical scale (Table 1, Q7) further confirmed these trends: out of 10 red was 6, blue was 5, and both green and neutral were 2.

### Statistical differences in glare perception between the four colored conditions

Descriptive results indicated that participants perceived glare differently across the colored conditions, despite these conditions exhibiting similar glare metric values. To evaluate the statistical significance of these differences, we performed a Friedman test, which revealed a highly significant effect of color on subjective glare perception (χ^2^ = 64.60, df = 3, *p* = 6.1 × 10⁻¹⁴), confirming that glare responses varied significantly across the four colored scenarios. Furthermore, we conducted another Friedman test on the subset of data excluding participants (9%) who were dissatisfied by the thermal conditions to ensure that our findings were not confounded, and we found that the results remained substantively unchanged (*p* < 0.01).

A post-hoc pairwise Dunn’s test with Bonferroni correction (p-values × 6) was conducted to explore these differences further where effect sizes and their interpretation are based on Cliff’s delta. As shown in Table 3, the mean differences between the glare responses were statistically significant with a large effect size for the following pairs: Neutral- Red (*p* < 0.001, Cliff’s delta = 0.53) and Green- Red (*p* < 0.001, Cliff’s delta = 0.57), with consistently higher glare ratings under Red glazing. The pairs Neutral-Blue and Green-Blue also had statistically significant differences in glare perception, but with a moderate effect size (*p* < 0.05, Cliff’s delta = 0.30). No statistically significant differences were found between the Neutral-Green and Blue-Red pairs. These inferential results corroborate the trends observed in participants’ glare ratings that red and blue glazing led to significantly greater discomfort than green and neutral ones.

**Table 3:** Results of pairwise comparisons in glare perception between the four colored conditions.

Group1	Group2	n1	n2	*p*-value	*p*-value (Bonferroni adjusted)	Effect size (Cliff’s delta)	Magnitude
Neutral	Green	53	52	0.951	1	0.05	small
Neutral	Blue	53	50	0.0025	0.015	0.30	moderate
Neutral	Red	53	50	8.4E-07	5.09E-06	0.53	large
Green	Blue	52	50	0.002	0.01	0.31	moderate
Green	Red	52	50	7.0E-07	4.0E-06	0.57	large
Blue	Red	50	50	0.056	0.339	0.23	small

## Discussions

### Spectral weighting in glare models

The findings of this study highlight a critical gap in current glare models as the discomfort glare was not perceived equally for conditions having similar glare metric values but different colors. Current glare models do not incorporate spectral characteristics of the glare source and only differentiate glare sources in terms of luminance values, derived using the CIE V_2°_(l) function. The V_2°_(l) function peaks in the mid-wavelength region at 555 nm and is least sensitive in the short- and long-wavelength regions. However, from our findings it’s clear that glare sources with similar luminance but dominant SPD in the short- or the long-wavelength regions induced highest discomfort, implying that V_2°_(l) is *not* accurately representing human eye’s spectral sensitivity when a high-intensity colored glare source is in the field of view. For that reason, the spectral weighting for glare evaluations in such situations needs modifications.

Furthermore, since our results of higher discomfort under red glazing depart from past studies that reported similar discomfort from white, green, and red LEDs of equal luminance but higher discomfort from blue LEDs^18–21^ (see Supplementary Table S1). As a result, all the previously proposed discomfort glare spectral sensitivity functions (V_DG_(l)^20^)- having a higher weighting in the short-wavelength region compared to V_2°_(l) but a similar weighting in the mid-and long-wavelength regions as V_2°_(l)- were not able to describe the perceived glare in our study, especially not those attained under the red conditions. A recent study^6^ comparing daylight glare from blue glazing to neutral, similarly showed that V_DG_(l) underestimates the increased glare magnitude of a blue sun disc. This inapplicability likely stems from very different visual environment in our experiments that include high adaptation levels, a much higher intensity glare source (the sun), and broader daylight spectra, whereas prior LED studies had very low ambient light (< 10 cd/m^2^) and narrow-band spectra.

A deeper concern is that luminance-based glare models assume perceptual additivity, by using spectral weighting functions such as V_2°_(l) or V_DG_(l) (several V_DG_(l) are compared by Yang et al. ^20^). However, human perception of brightness is non-additive except in the narrow experimental conditions used to derive such functions^67,68^. In the case of brightness, this additivity failure means that accurate measures of brightness must include an additional chromatic signal in a phenomenon known as the Helmholt-Kohlrausch (H-K) effect^69,70^- where equiluminant stimuli do not appear equally bright to observers if they vary in chromatic saturation and hue. Findings from this study suggest that discomfort glare, like brightness, may also be influenced by both achromatic and chromatic signals.

To account for H-K effect, Color Appearance Models (CAMs), which mathematically estimate early stages of visual processing (cone response, adaptation, compression, and opponency), may provide a better foundation for glare prediction. Recent work by Yang et al. introduced QUGR, a glare metric incorporating the CAM15u model for colored electric light^20^. In addition to CAMs, the CIE 200:2011 Supplementary System of Photometry^71^ for colored lighting also incorporate the H-K effect. However, this system has, to our knowledge, never been tested for glare scenarios. In the next section, we evaluate both CAM- based and CIE 200:2011 approaches for our dataset to assess their applicability in predicting discomfort glare from colored daylight sources.

### Incorporating H-K effect in glare models

#### Applying color appearance model

Glare metrics based on brightness predictions from CAMs which account for the H-K effect offer the possibility of predicting glare from chromatic sources where metrics based solely on luminance would fail. We tested three such models that incorporate mechanisms to account for brightness perception failures under chromatic conditions : CAM15u (following the QUGR approach^20^, CAM18sl^72^, and a revised version of CIECAM16^70,73^. Each model reflects a distinct visual context: (i) CAM15u^74^ was designed to predict the appearance of unrelated visual stimuli, such as colored lights viewed in a dark environment, (ii) CAM18sl^72^ extended CAM15u to include self-luminous stimuli with a gray background of variable luminance, (iii) CIECAM16^75^ is a general-purpose model which recently been extended (CAM16-Hellwig) to include the H-K effect^70,73^.

One key obstacle in using these models is that the luminance of the sun exceeds the range in which CAM18sl and CIECAM16 are well behaved. Specifically, the Michaelis-Menten functions that represent the non-linear cone response compression in both models become unreliable around 10^4^ cd/m^2^ and 10^6^ cd/m^2^, respectively. This leads to artificial suppression of colorfulness predictions and, consequently, underestimated brightness differences between colored glazing. As this is a mathematical artifact rather than a perceptual effect, further refinement of these models is necessary for glare prediction of very high-luminance stimuli. However, since all the test conditions had similar luminance across four colors, we use a normalized values of 1000 cd/m² to keep the predictions within the valid range of the models for testing purpose.

The main inputs into each model are either CIE LMS or CIE XYZ values of the sun seen through each colored-glazing that were measured during the experiments. These values were then scaled to give them a luminance of 1000 cd/m^2^, thus maintaining the chromaticity (cone response ratio) of each stimulus while keeping the inputs within the functional range of the models. A reference white input for CIECAM16 was set to the average measured color of the sun seen through the neutral glazing at 1000 cd/m^2^. CAM15u requires a background color input, which—following the common “gray world” assumption—was set to the same color at 200 cd/m^2^ (the results were not sensitive to changes in these assumed luminance values). Additionally, in CIECAM16, the degree of chromatic adaptation was set to 1 and the surround conditions were set to “average”.

**Fig. 6:**
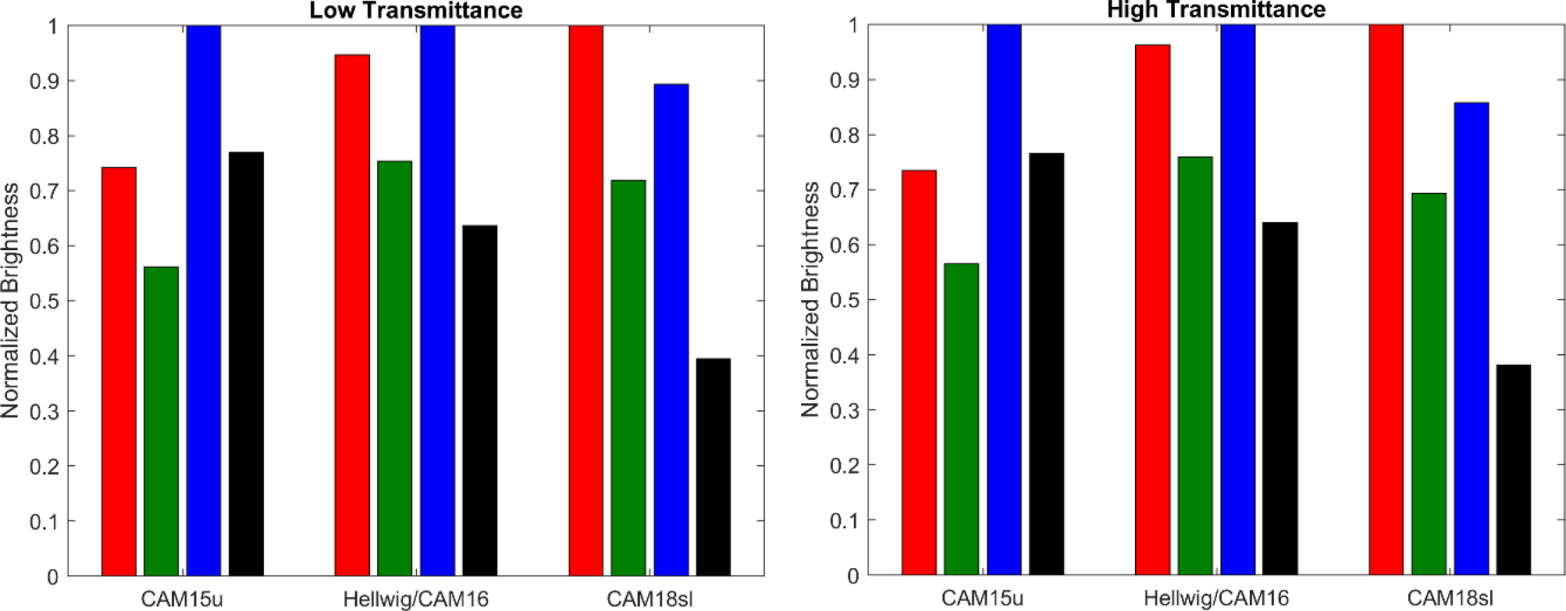
Normalized brightness of the sun seen through the red, green, blue and neutral glazing as predicted by three color appearance models where the model input values were scaled to keep within the operational range of the CAMs.

The resulting predicted brightness of the sun seen through each filter is shown in Fig. 6. Both CAM18sl and CIECAM16 predict that the sun will appear much brighter through the red and blue glazing than through the green and neutral, which is in alignment with the subjective responses. Given that the sun had similar luminance, the differences in brightness are directly caused by the differences in the predicted colorfulness of colored sun disc. The slight differences between the two models are due to differences in how colorful they predict each stimulus to be, owing to models’ slightly different methods of predicting colorfulness as a function of hue. However, the brightness predictions of CAM15u disagree with the subjective responses, predicting that the red and neutral glazing are equally bright and green is the least bright. This seeming error can be attributed to the lack of chromatic adaptation in CAM15u. The sun seen through the neutral glazing should have a very low colorfulness, but CAM15u predicts the sun to be equally colorful when seen through neutral and red glazing. This indicates that the default white point of CAM15u does not match the state of chromatic adaptation of the observers in this experiment.

This analysis revealed that CAM18sl and CAM16-Hellwig show promise for glare prediction, suggesting that incorporating chromatic input into brightness perception maybe more effective for predicting glare from colored daylight sources than modifying the spectral weighting function alone. However, their ability to predict the H-K effect is limited to lower luminance ranges, therefore, further development is needed to have models that are robust in all glare applications.

#### Applying CIE 200:2011 supplementary photometry

The CIE 200:2011 Supplementary System of Photometry^71^ estimates ‘equivalent luminance’ by incorporating chromaticity (CIE xy) to account for the spectral contribution of colored light sources. We calculated a modified version of equivalent luminance by using an equal energy white point as a reference instead of 555 nm monochromatic light as specified in CIE 200:2011. The CIE xy input was derived from the spectroradiometer measurements, and the resulting equivalent luminance values were used to adjust DGP.

**Fig. 7:**
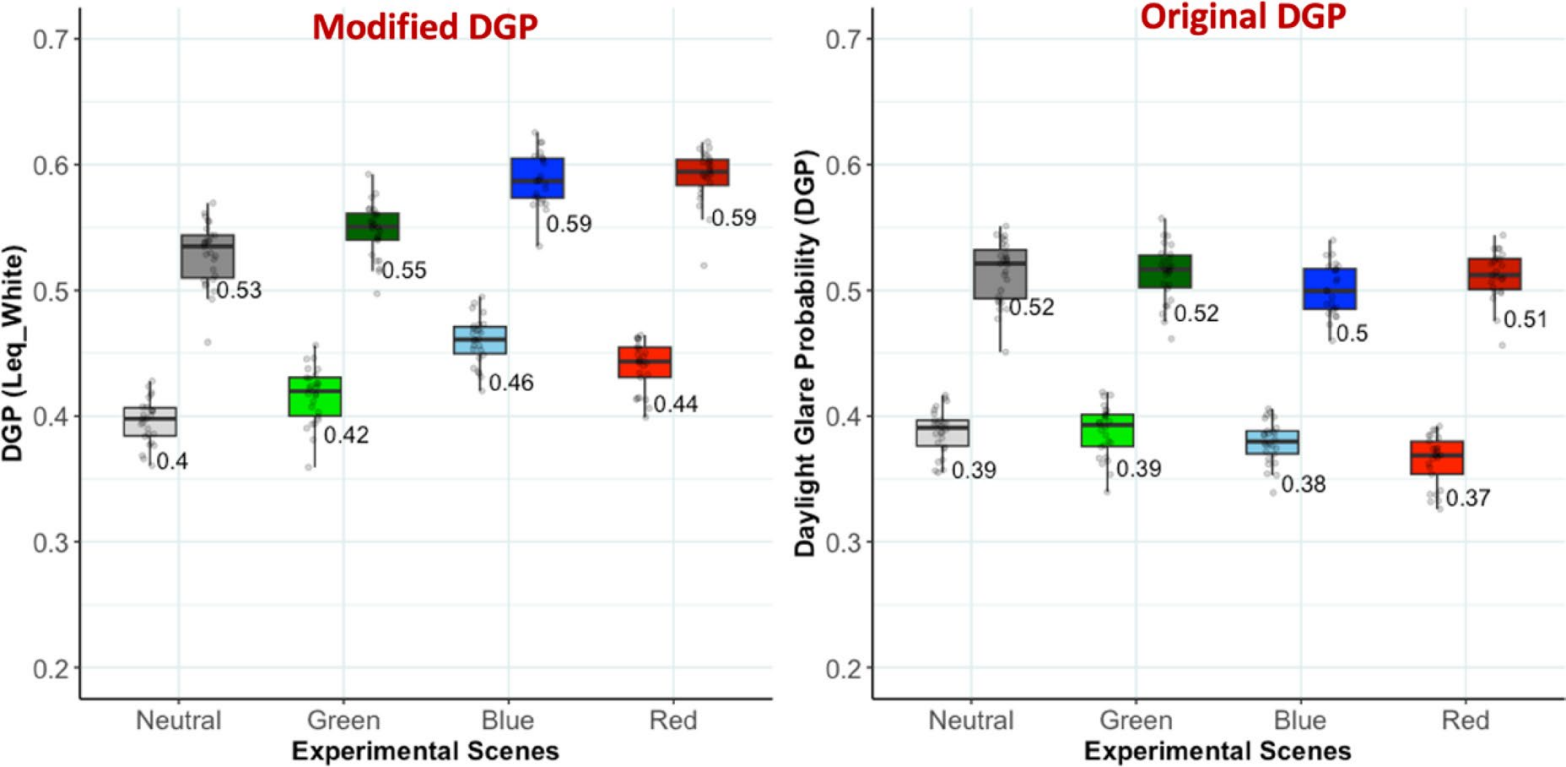
Modified DGP when using Equivalent luminance from CIE 200:2011 (left), compared to original DGP when using standard V(l) based luminance (right).

As shown in Fig. 7, the modified DGP aligns more closely with participants’ subjective glare responses, especially for blue and red glazing, by predicting higher discomfort than for neutral and green. However, it slightly overestimates glare from blue relative to red, differing from participants’ responses (Fig. 5). Since the stimuli variations in terms of DGP values are very small, a direct comparison of correlations between the original and modified values is not appropriate. Nevertheless, the modified model trends in right direction when compared to subjective responses. Although not fully capturing the distinction as the magnitude of modified DGP differences between colored conditions does not always align with participants’ glare responses. Additional data and a broader range of stimuli is needed to assess the suitability of supplementary photometry for glare from colored daylight sources.

### Exploratory analysis of gaze behaviour during the experiments

As a brief exploratory step, we investigated whether the glazing color influenced participants’ gaze behavior, motivated by the research in color psychology that suggest colors can evoke varying attention and arousal levels, potentially affecting visual focus. For example, red is often associated with increased arousal and heightened attention, while blue may promote a calming effect and reduce visual engagement^76,77^. To examine this, we extracted gaze data by processing video recordings of the participants’ faces with the OpenFace^43^ tool. However, due to limitations of tool in predicting gaze in certain viewing angles required discarding ~ 52% of the data. In the remaining dataset (*N* ~ 25 per condition), we found no clear differences in gaze patterns between the four colored scenes (Supplementary Figure S4). Although preliminary, this analysis highlights the potential value of further exploration with more robust eye-tracking methods and larger samples to understand how color influences visual attention and glare perception.

### Study limitations

The main limitations of this study, which may impact the generalizability and accuracy of the results, include the following:

- Results are specific to the filtered daylight spectra tested and may differ with variations in glazing properties, climate, or geographic location.
- This study only tested a limited range of saturated colored glazing. Future studies should test less saturated and more commercially representative glazing options across a broader color range.
- The experiment included only two transmittance levels (high and low), limiting the range of luminance and glare metric values. This constrained our ability to fully evaluate CAMs and CIE 200:2011 based glare model performance using correlation metrics. Therefore experiments with wider variations in glare intensity and color are required to determine whether tested model can be modified or a new model is needed.
- This study was limited to a single desk position with sun in the central field of view, which, while optimal for evaluating worst-case glare scenario, is not representative of typical office setups. Since color perception varies across the visual field^78^, results may differ with other viewing positions.
- The participant sample included only healthy adults aged 18–30, not representative of broader workplace demographics. As spectral sensitivity changes with age^79^, the findings may not extend to older adults or those with vision impairments.

## Conclusions

This study evaluated the effect of the color of sun disc filtered through saturated-colored glazing on discomfort glare perception. Participants were exposed to four conditions that had similar sun luminance, a similar apparent sun position relative to their FOV, and similar E_v_ but varied in the color of sun disc because of its filtering through red, green, blue, or neutral glazing. We found that color of sun disc has a strong influence of glare perception despite similar photometric conditions, with red and blue conditions inducing more frequent reports of glare than neutral and green ones. While increased discomfort under blue conditions aligns with prior studies with colored LEDs, our most striking result was the unexpectedly high level of discomfort induced by the red glazing which is contrary to the existing works that has not linked red LEDs to increased discomfort. This finding challenges the current understanding of visual comfort and suggests that the mechanism driving the glare perception are not fully captured by current models.

These findings also confirm that existing glare models based on V(λ) weighting do not adequately predict glare in such conditions. Color appearance models (CAM18sl and CAM16-Hellwig) and CIE 200:2011 offer improved predictions by incorporating the H-K effect, reinforcing that perceived brightness varies with chromatic saturation. However, CAMs require further development to perform reliably at high luminance levels typical of daylight glare. Similarly, while CIE 200:2011 incorporates color, its applicability in terms of quantifiability remains to be fully validated. Future work should investigate a broader range of stimuli in terms of intensity and spectrum to establish a more comprehensive understanding, and whether glare perception is driven more by glare source’s SPD or by perceived color. This is critical to determine whether the current findings could be extended to colored façades having similar color appearances but varying SPDs and vice versa.

An important practical takeaway of this study is that discomfort glare can likely be minimized by avoiding saturated red and blue colored glazing. These results can provide guidance for more nuanced development goals for EC glazing coatings, colored glazing, and colored building-integrated photovoltaics façades, where color and comfort must be balanced.

## Supplementary Information

Below is the link to the electronic supplementary material.

Supplementary Material 1

## Data Availability

Data is provided within the manuscript and supplementary material file. Raw data is available from the corresponding author upon request.

## References

[1] Boyce, P. Light, lighting and human health. *Lighting Res. Technol.* **54**, 101–144 (2022).

[2] Farley, K. M. J. & Veitch, J. A. A Room with a View: A Review of the Effects of Windows on Work and Well-Being. 10.4224/20378971 (2001).

[3] Heschong, L., Wright, R. L. & Okura, S. Daylighting impacts on human performance in school. *J. Illum. Eng. Soc.* **31**, 101–114 (2002).

[4] Jamrozik, A. et al. Access to daylight and view in an office improves cognitive performance and satisfaction and reduces eyestrain: A controlled crossover study. *Build. Environ.* **165**, 106379 (2019).

[5] Jain, S., Karmann, C. & Wienold, J. Behind electrochromic glazing: assessing user’s perception of glare from the sun in a controlled environment. *Energy Build.* **256**, 111738 (2022).

[6] Jain, S., Wienold, J., Lagier, M., Schueler, A. & Andersen, M. Perceived glare from the sun behind tinted glazing: comparing blue vs. color-neutral tints. *Build. Environ.* **234**, 110146 (2023).

[7] Wienold, J., Jain, S. & Andersen, M. Transmittance thresholds of electrochromic glazing to achieve annual low-glare work environments. In *E3S Web of Conferences* vol. 362 08001EDP Sciences, (2022).

[8] Baetens, R., Jelle, B. P. & Gustavsen, A. Properties, requirements and possibilities of smart windows for dynamic daylight and solar energy control in buildings: A state-of-the-art review. *Sol. Energy Mater. Sol. Cells*. **94**, 87–105 (2010).

[9] Arsenault, H., Hébert, M. & Dubois, M. C. Effects of glazing colour type on perception of daylight quality, arousal, and switch-on patterns of electric light in office rooms. *Build. Environ.* **56**, 223–231 (2012).

[10] Chen, X., Zhang, X. & Du, J. Glazing type (colour and transmittance), daylighting, and human performances at a workspace: A full-scale experiment in Beijing. *Building Environ.* (2019).

[11] Chinazzo, G., Wienold, J. & Andersen, M. Combined effects of daylight transmitted through coloured glazing and indoor temperature on thermal responses and overall comfort. *Build. Environ.*10.1016/j.buildenv.2018.08.045 (2018).

[12] Liang, R., Kent, M., Wilson, R. & Wu, Y. Development of experimental methods for quantifying the human response to chromatic glazing. *Build. Environ.*10.1016/j.buildenv.2018.09.044 (2018).

[13] Liang, R., Kent, M., Wilson, R. & Wu, Y. The effect of thermochromic windows on visual performance and sustained attention. *Energy Build.* **236**, 110778 (2021).

[14] Flannagan, M. J. *Subjective and Objective Aspects of Headlamp Glare: Effects of Size and Spectral Power Distribution*. 19 (1999).

[15] Flannagan, M. J., Sivak, M., Ensing, M. & Simmons, C. J. *Effect of Wavelength on Discomfort Glare from Monochromatic Sources.* http://deepblue.lib.umich.edu/handle/2027.42/64064 (1989).

[16] CIE 086-1990. *CIE 1988 2° Spectral Luminous Efficiency Function for Photopic Vision | CIE*. https://cie.co.at/publications/cie-1988-2-spectral-luminous-efficiency-function-photopic-vision (1988).

[17] Sivak, M., Schoettle, B., Minoda, T. & Flannagan, M. J. Short-Wavelength content of LED headlamps and discomfort glare. *J. Illuminating Eng. Soc.* **2**, 145–154 (2005).

[18] Fekete, J., Sik-Lányi, C. & Schanda, J. Spectral discomfort glare sensitivity investigations. *Ophthalmic Physiol. Opt.* **30**, 182–187 (2010). 20444123 10.1111/j.1475-1313.2009.00696.x

[19] Bullough, J. D. Spectral sensitivity for extrafoveal discomfort glare. *J. Mod. Opt.* **56**, 1518–1522 (2009).

[20] Yang, Y., Luo, R. M. & Huang, W. J. Assessing glare, part 3: glare sources having different colours. *Lighting Res. Technol.* **1477153516676640**10.1177/1477153516676640 (2016).

[21] Kimura-Minoda, T. & Ayama, M. Evaluation of discomfort glare from color leds and its correlation with individual variations in brightness sensitivity. *Color. Res. Application*. **36**, 286–294 (2011).

[22] Sweater-Hickcox, K., Narendran, N., Bullough, J. & Freyssinier, J. Effect of different coloured luminous surrounds on LED discomfort glare perception. *Lighting Res. Technol.* **45**, 464–475 (2013).

[23] Niedling, M. & Völker, S. Influence of a glare sources spectrum on discomfort glare – a physiological explanation for a psychological phenomenon. In *proceedings of the conference at the cie midterm meeting 2017 23–25 october* jeju, republic of korea 866–870 (International Commission on Illumination, CIE, Jeju Island, Republic of Korea, 2018). 10.25039/x44.2017.PO21 (2017).

[24] Bullough, J. D., Van Derlofske, J., Dee, P., Chen, J. & Akashi, Y. An investigation of headlamp glare: intensity, spectrum and size. (2004).

[25] Wei, M. et al. Field study of office worker responses to fluorescent lighting of different CCT and lumen output. *J. Environ. Psychol.* **39**, 62–76 (2014).

[26] Chen, P. L. et al. A portable inspection system to estimate direct glare of various LED modules. In (eds. Asundi, A. K. & Fu, Y.) 95241X (Singapore, Singapore, 10.1117/12.2189599 (2015).

[27] Zhang, J. et al. P.30: Effect of the Correlated Color Temperature of Light on Overhead Glare in Offices. *SID Symposium Digest of Technical Papers* 44, 1096–1098 (2013).

[28] Coloured, B. I. P. V. *Market, Research and Development*. https://iea-pvps.org/wp-content/uploads/2020/01/IEA-PVPS_15_R07_Coloured_BIPV_report.pdf (2019).

[29] Hirschl, B. *Acceptability of Solar Power Systems a Study on Acceptability of Photovoltaics with Special Regard To the Role of Design* (IÖW, 2005).

[30] Woo, J., Moon, S. & Choi, H. Economic value and acceptability of advanced solar power systems for multi-unit residential buildings: the case of South Korea. *Appl. Energy*. **324**, 119671 (2022).

[31] Polo López, C. S., Troia, F., Nocera, F. & Photovoltaic, B. I. P. V. Systems and architectural heritage: new balance between conservation and Transformation. An assessment method for heritage values compatibility and energy benefits of interventions. *Sustainability* **13**, 5107 (2021).

[32] Cannavale, A., Ayr, U. & Martellotta, F. Energetic and visual comfort implications of using perovskite-based building-integrated photovoltaic glazings. *Energy Procedia*. **126**, 636–643 (2017).

[33] Gratzel, M. & O’Regan, B. A low-cost, high-efficiency solar cell based on dye-sensitized colloidal TiO2 films. *Nature* **353**, 737–740 (1991).

[34] Jain, S., Karmann, C. & Wienold, J. Subjective assessment of visual comfort in a daylit workplace with an electrochromic glazed façade. *J. Phys. : Conf. Ser.* **2042**, 012179 (2021).

[35] Charness, G., Gneezy, U. & Kuhn, M. A. Experimental methods: Between-subject and within-subject design. *J. Econ. Behav. Organ.* **81**, 1–8 (2012).

[36] Faul, F., Erdfelder, E., Buchner, A. & Lang, A. G. Statistical power analyses using G*Power 3.1: tests for correlation and regression analyses. *Behav. Res. Methods*. **41**, 1149–1160 (2009). 19897823 10.3758/BRM.41.4.1149

[37] Ishihara, S. Handaya, Tokyo, Hongo Harukicho,. Tests for color-blindness. (1917).

[38] Farnsworth, D. *The Farnsworth Dichotomous Test for Color Blindness: Panel D-15; Manual* (The Psychological Corp, 1947).

[39] Horne, J. A. & Östberg, O. A. Self-Assesment questionnaire to determine Morningness-Eveningness in human circadian rhythms. *Int. J. Chronobiology*. **4**, 97–110 (1976). 1027738

[40] Kent, M., Altomonte, S., Tregenza, P. R. & Wilson, R. Temporal variables and personal factors in glare sensation. *Lighting Res. Technol.* **48**, 689–710 (2015).

[41] Pierson, C., Wienold, J. & Bodart, M. Review of factors influencing discomfort glare perception from daylight. *LEUKOS* **0**, 1–38 (2018).

[42] Kassner, M., Patera, W. & Bulling, A. Pupil: an open source platform for pervasive eye tracking and mobile gaze-based interaction. in *Proceedings of the ACM International Joint Conference on Pervasive and Ubiquitous Computing: Adjunct Publication* 1151–1160 (Association for Computing Machinery, New York, NY, USA, 2014). 1151–1160 (Association for Computing Machinery, New York, NY, USA, 2014). 10.1145/2638728.2641695 (2014).

[43] Baltrusaitis, T., Zadeh, A., Lim, Y. C. & Morency, L. P. OpenFace 2.0: facial behavior analysis toolkit. *2018 13th IEEE Int. Conf. Automatic Face Gesture Recognit. (FG 2018)*. **59-66**10.1109/FG.2018.00019 (2018).

[44] CEN. Light And Lighting - Lighting Of Work Places - Part 1: Indoor Work Places; German Version EN 12464-1. (2021).

[45] Nichols, A. L. & Maner, J. K. The Good-Subject effect: investigating participant demand characteristics. *J. Gen. Psychol.* **135**, 151–166 (2008). 18507315 10.3200/GENP.135.2.151-166

[46] Jain, S. et al. Influence of macular pigment on the sensitivity to discomfort glare from daylight. *Sci. Rep.* **13**, 18551 (2023). 37899478 10.1038/s41598-023-45785-x PMC10613614

[47] Text Analysis Tool. *Text Anal. Tools* https://app.readable.com/text/ (2020).

[48] Osterhaus, W. & Bailey, I. L. Large Area Glare Sources and Their Effect on Discomfort and Visual Performance at computer Workstations. in *Proceedings of the IEEE Industry Applications Society Annual Meeting* 1825–1829 (Houston, TX, USA, 1992). 1825–1829 (Houston, TX, USA). 10.1109/Ias.1992.244537 (1992).

[49] Pierson, C. *Discomfort Glare Perception from Daylight: Influence of the socio-environmental Context* (Université catholique de Louvain, Louvain-la-Neuve, 2019).

[50] Chinazzo, G. Daylight and temperature in buildings: interaction effects on human responses. (Ecole polytechnique fédérale de Lausanne, Lausanne, Switzerland, (2019).

[51] Fotios, S. Research methods to avoid bias in categorical ratings of brightness. *Leukos* **5**, 167–181 (2009).

[52] Wienold, J. & Andersen, M. Evalglare 2.0: new features, faster and more robust HDR-image evaluation. in 3rd International Radiance Workshop (Padova, Italy, (2016).

[53] Mansencal, T. et al. Colour 0.4.2. Zenodo 10.5281/zenodo.7367239 (2022).

[54] Cronbach, L. J. Coefficient alpha and the internal structure of tests. *Psychometrika* **16**, 297–334 (1951).

[55] Friedman, M. The use of ranks to avoid the assumption of normality implicit in the analysis of variance. *J. Am. Stat. Assoc.* **32**, 675–701 (1937).

[56] Dunn, O. J. Multiple comparisons using rank sums. *Technometrics* **6**, 241–252 (1964).

[57] Cabin, R. & Mitchell, R. To bonferroni or not to bonferroni: when and how are the questions. *Bull. Ecol. Soc. Am.* **81**, 246–248 (2000).

[58] Field, A., Miles, J. & Field, Z. *Discovering Statistics Using R* (SAGE Publications, Inc, 2012).

[59] Garretón, J. Y., Rodriguez, R. & Pattini, A. Effects of perceived indoor temperature on daylight glare perception. *Building Res. Inform.* **0**, 1–14 (2015).

[60] International Commission On Illumination (CIE). Lighting of work places - Part 1: Indoor. (2002).

[61] Zhang, J., Lv, K., Zhang, X., Ma, M. & Zhang, J. Study of human visual comfort based on sudden vertical illuminance changes. *Buildings* **12**, 1127 (2022).

[62] Wienold, J. et al. Cross-validation and robustness of daylight glare metrics. *Lighting Res. Technol.* **1477153519826003**10.1177/1477153519826003 (2019).

[63] Karmann, C. et al. User assessment of fabric shading devices with a low openness factor. *Build. Environ.* **109707**10.1016/j.buildenv.2022.109707 (2022).

[64] Konstantzos, I. & Tzempelikos, A. Daylight glare evaluation with the sun in the field of view through window shades. *Build. Environ.* **113**, 65–77 (2017).

[65] Pierson, C., Piderit, B., Iwata, T., Bodart, M. & Wienold, J. Is there a difference in how people from different socio-environmental contexts perceive discomfort due to glare from daylight? *Lighting Res. Technol.* **1477153520983530**10.1177/1477153520983530 (2021).

[66] Quek, G. et al. Comparison of questionnaire items for discomfort glare studies in daylit spaces. *Lighting Res. Technol.* **55**, 730–758 (2023).

[67] Kaiser, P. K. & Wyszecki, G. Additivity failures in heterochromatic brightness matching. *Color. Res. Application*. **3**, 177–182 (1978).

[68] Wagner, G. & Boynton, R. M. Comparison of four methods of heterochromatic photometry. *J. Opt. Soc. Am. JOSA*. **62**, 1508–1515 (1972). 10.1364/josa.62.001508 4643012

[69] Hellwig, L., Stolitzka, D., Fairchild, M., Stolitzka, D. & Fairchild, M. Why achromatic response is not a good measure of brightness. *Color. Imaging Conf.* **30**, 1–5 (2022).

[70] Hellwig, L., Stolitzka, D. & Fairchild, M. D. The brightness of chromatic stimuli. *Color. Res. Application*. **49**, 113–123 (2024).

[71] CIE. *CIE Supplementary System of Photometry*. https://cie.co.at/publications/cie-supplementary-system-photometry (2011). 10.1111/j.1475-1313.2006.00357.x 16684150

[72] Hermans, S., Smet, K. G. & Hanselaer, P. Color appearance model for self-luminous stimuli. *J. Opt. Soc. Am. JOSAA*. **35**, 2000–2009 (2018). 10.1364/JOSAA.35.002000 30645289

[73] Hellwig, L. & Fairchild, M. D. Brightness, lightness, colorfulness, and chroma in CIECAM02 and CAM16. *Color. Res. Application*. **47**, 1083–1095 (2022).

[74] Withouck, M., Smet, K. A. G., Ryckaert, W. R. & Hanselaer, P. Experimental driven modelling of the color appearance of unrelated self-luminous stimuli: CAM15u. *Opt. Express OE*. **23**, 12045–12064 (2015). 10.1364/OE.23.012045 25969293

[75] , C. I. E. The CIE 2016 colour appearance model for colour management systems: CIECAM16. *CIE JTC 10*. **248**10.25039/TR.248.2022 (2022).

[76] Elliot, A. J. & Maier, M. A. Color psychology: effects of perceiving color on psychological functioning in humans. *Ann. Rev. Psychol.* **65**, 95–120 (2014). 23808916 10.1146/annurev-psych-010213-115035

[77] Kaya, N. & Epps, H. H. Relationship between color and emotion: A study of college students. *Coll. Student J.* **38**, 396–405 (2004).

[78] Hansen, T., Pracejus, L. & Gegenfurtner, K. R. Color perception in the intermediate periphery of the visual field. *J. Vis.* **9**, 26 (2009). 10.1167/9.4.26 19757935

[79] Curcio, C. A., Medeiros, N. E. & Millican, C. L. Photoreceptor loss in age-related macular degeneration. *Invest. Ophthalmol. Vis. Sci.* **37**, 1236–1249 (1996). 8641827

