# Supplementary material for: Influence of color on glare perception revealed when seeing the sun through colored glazing
Source: Sci Rep. 2025 Oct 14;15:34124. doi: 10.1038/s41598-025-21737-5 (PMC12521605; doi:10.1038/s41598-025-21737-5)
Supplement: Supplementary file 1 — Supplementary Material 1 [file 41598_2025_21737_MOESM1_ESM.docx]

# Supplementary Information

**Supplementary Table S1**: A literature review of previous studies on the influence of color of light on discomfort glare

| **Study** | **Sample size** | **Type of light source** | **Tested light sources’ color/dominant wavelength/CCT** | **Angle between source & observer** | **Glare assessment method** | **Key Results** |
| --- | --- | --- | --- | --- | --- | --- |
| (Jain, Wienold, Lagier, et al., 2023)^1^ | 75 | Daylight | Color-neutral glazing, blue glazing (peak wavelength 455 nm) | 32°-25° | 4-point ordinal scale | Participants perceived glare more strongly under blue glazing compared to color-neutral glazing. |
| (Suzuki et al., 2019)^2^ | 23 | LCD screen | Black, blue, cyan, green, yellow, red, magenta | 4.62° | Pupil size, side by side luminance matching | Blue was rated as brightest condition. Blue hues constricted pupil more than other hues |
| (Niedling & Völker, 2018)^3^ | 36 | LED | 3700 K, 3400 K, 6500 K, (having same Ev) | 4° vertical | 9-point category scale | LED with 6500 K CCT showed higher glare ratings than all other LEDs |
| (Choi & Ko, 2018)^4^ | 33 | LED | 2700 K, 3000 K, 4000 K, 5000 K, 6000 K | overhead | 7-point category scale | No difference in glare perception |
| (Yang et al., 2016)^5^ | 20 | LED | Blue1-435 nm, Blue2-455 nm, Blue3-477 nm, Green-527 nm, Red-623 nm, White-4200 K | 0°, 20° | 7-point category scale | Blue (Blue1>Blue2>Blue3) LED was most discomforting, then red, green and white LEDs |
| (P.-L. Chen et al., 2015)^6^ | 8 | LED | 3100 K, 4000 K, 5300 K | 0°, 10° | De Boer 9-pt scale | Higher CCT caused higher discomfort |
| (Wei et al., 2014)^7^ | 26 | Fluorescent lighting | 5000 K, 3500 K | overhead | 7-point agreement scale | Higher CCT caused higher discomfort |
| (Zhang et al., 2013)^8^ | 18 | Fluorescent lighting | 4000 K, 6300 K | overhead | 7-point category scale | Higher CCT caused higher discomfort |
| (Sweater-Hickcox et al., 2012)^9^ | 10 | LED | Glare source: White LED (6500 k), Background1: Yellow LED (Green-525 nm+ Red-635 nm), Background2: Blue-465 nm | 2° | De Boer 9-point scale | Blue background LEDs were rated more glary than yellow or white |
| (Kimura-Minoda & Ayama, 2010)^10^ | 15 | LED, tungsten-halogen | Red1-628 nm, Red2-620 nm, Green-542 nm, Blue-459 nm, Amber-586 nm, red tungsten-halogen bulb-617 nm, white-6800 K | 2°, 10° | De Boer 9-pt scale | Blue had highest glare perception. No significant difference among other stimuli. Brightness and glare perception were correlated |
| (Fekete et al., 2009)^11^ | 10 | Xenon lamp | 420 nm-630 nm at 10 nm steps | 2°, 10° | De Boer 9-pt scale | Higher discomfort under shorter wavelength |
| (Bullough, 2009)^12^ | 24 | Xenon lamp | 450 nm, 510 nm, 590 nm, 650 nm and 700 nm | 5°, 10° | De Boer 9-pt scale | Higher discomfort under shorter wavelength |
| (Sivak, 2005)^13^ | 12 | LED | 4000 K, 4800 K, 6600 K | 0.5° | De Boer 9-pt scale | Discomfort glare was linearly related to amount of blue content in LED |
| (Bullough et al., 2004)^14^ | 31 | HID, halogen, blue-filtered halogen lamp | 450 nm, 510 nm, 590 nm, 650 nm, 700 nm, 420 nm, 450 nm, 490 nm, 577nm | 5°, 10° | De Boer 9-pt scale | Higher discomfort under shorter wavelength. V(λ) does not accurately characterize discomfort glare. |
| (Flannagan, 1999)^15^ | 12 | Tungsten-halogen (TH) and HID | HID: blue-white, TH: yellow | 0.3°, 0.6° | Linear glare scale | SPD affected glare perception, HID were more discomforting than TH lamps |
| (Berman et al., 1995)^16^ | 12 | Fluorescent lighting | Cool-white, greenish blue-505 nm | 24°x33° | Visual analog scale (0 to 100mm) | Scotopically enhanced light source caused lower level of discomfort |
| (Flannagan et al., 1989)^17^ | 16 | Monochromatic lamps | 480 nm, 505 nm, 550 nm, 577 nm, 600 nm, 650 nm | 7° | De Boer 9-pt scale | Least comfortable- 480 nm > 505 nm > 650 nm > 600 nm > 550 nm > 577 nm-Most comfortable |

### **Supplementary Method**

### **Glazing selection criteria and properties**

To achieve the study objectives, we selected the glazing transmittance and color with pre-defined criteria which are described below. A series of pre-test measurements with HDR imaging was conducted to reach the final eight experimental conditions fulfilling our criteria.

The criteria for selecting the three saturated colored glazings (of blue, green, and red colors) were to have a minimum overlap between their respective peaks in spectral transmittance, covering distinct parts of the visible spectrum. The color-neutral glazing was chosen as a reference scenario to compare against the three colors since it is the most widely used glazing. After several iterations of ordering and measuring many colored films from different manufacturers with a goal to have same visible transmittances, we were able to approximately match the visible light transmittances τ_vis_ (that uses V(λ) for the spectral weighting ^18^ ) of four colored films in two groups of low and high transmittance within acceptable differences (maximum relative difference between the transmittance is 11%) and with the desired spectral transmittances. Supplementary Figure S1 plots the color of the glazing using CIE xy chromaticity coordinates against D65 standard illuminant.

The criteria for selecting two glazing transmittance levels (high and low) were to evaluate whether the effect of color could be observed at different glare source luminance levels. The two transmittance levels adopted for the glazings were, on average, 2.5% for the high-level glazings and 0.37% for the low-level ones. To create the colored sun window, we applied colored films between two transparent acrylic panels (τ_v_ =95%) and manually attached the panels to the existing fixed window glazing (τ_v_ =79%) facing the sun. We measured the spectral transmittance of each glazing unit (combination of colored filter and fixed window) and their angular behavior in a laboratory setup described by Steiner et. al ^19^. The measurement uncertainty of normal-hemispherical transmittance (τ_v,n-h_) is estimated to be less than 0.001. Supplementary Table S2 demonstrate the measured τ_v,n-h_ of all the glazing used in experiments. Supplementary Figure S2 shows the falsecolor luminance images of the two example scenes of high and low conditions that were experienced by the participants (images captured at eye level).

**Supplementary Table S2** Visible light transmittance and CIE xy chromaticity coordinates of window glazing used in the experiment

| **Glazing** | **Visible light Transmittance (τ_v,n-h_) weighted over V(λ)** | **CIE *xy* chromaticity coordinates** | |
| --- | --- | --- | --- |
|  |  | **x** | **y** |
| Blue_low | 0.39% | 0.14 | 0.06 |
| Green_low | 0.40% | 0.32 | 0.62 |
| Red_low | 0.33% | 0.69 | 0.29 |
| Neutral_low | 0.38% | 0.31 | 0.35 |
| Blue_high | 2.25% | 0.15 | 0.06 |
| Green_ high | 2.67% | 0.32 | 0.62 |
| Red_ high | 2.48% | 0.69 | 0.29 |
| Neutral_ high | 2.37% | 0.31 | 0.35 |
| View windows | 8.28% | 0.33 | 0.35 |

**Supplementary Figure S1**: CIE xy chromaticity coordinates of the blue, green, neutral and red glazing used in experiments


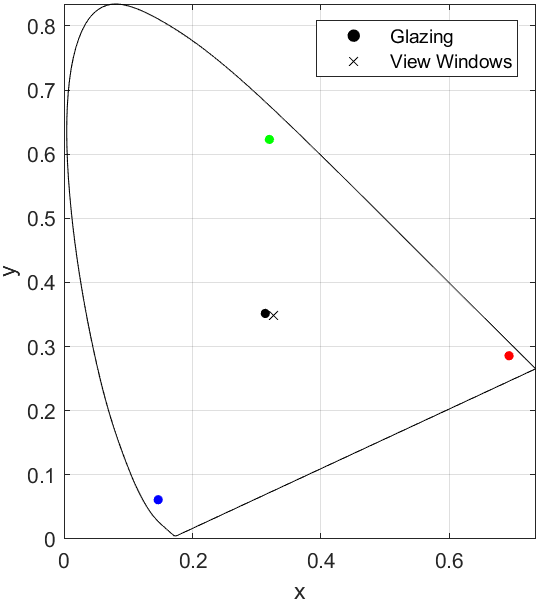


**Supplementary Figure S2**: Example of Falsecolor fisheye luminance images of the high and low-intensity conditions experienced by the participants with highest luminance value of the sun disc written on the respective images.


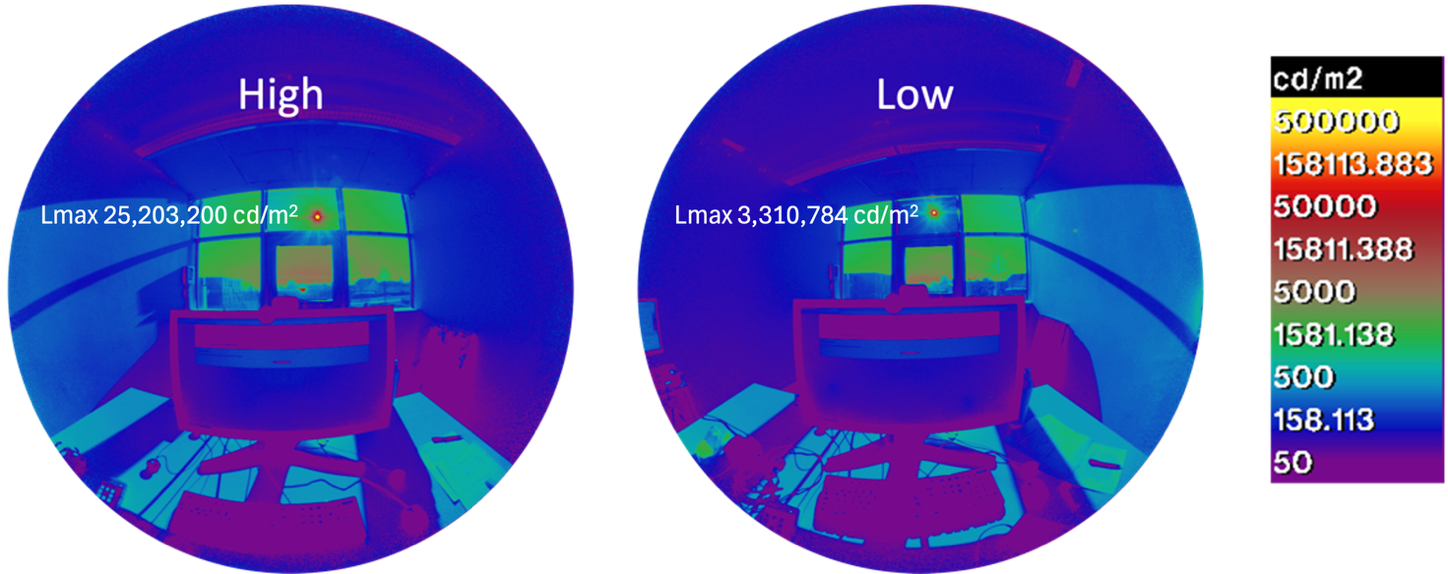


**Supplementary Figure S3:** Distribution of participants’ responses for the question “At this precise moment, how are you feeling?**”** compared across the four colored conditions


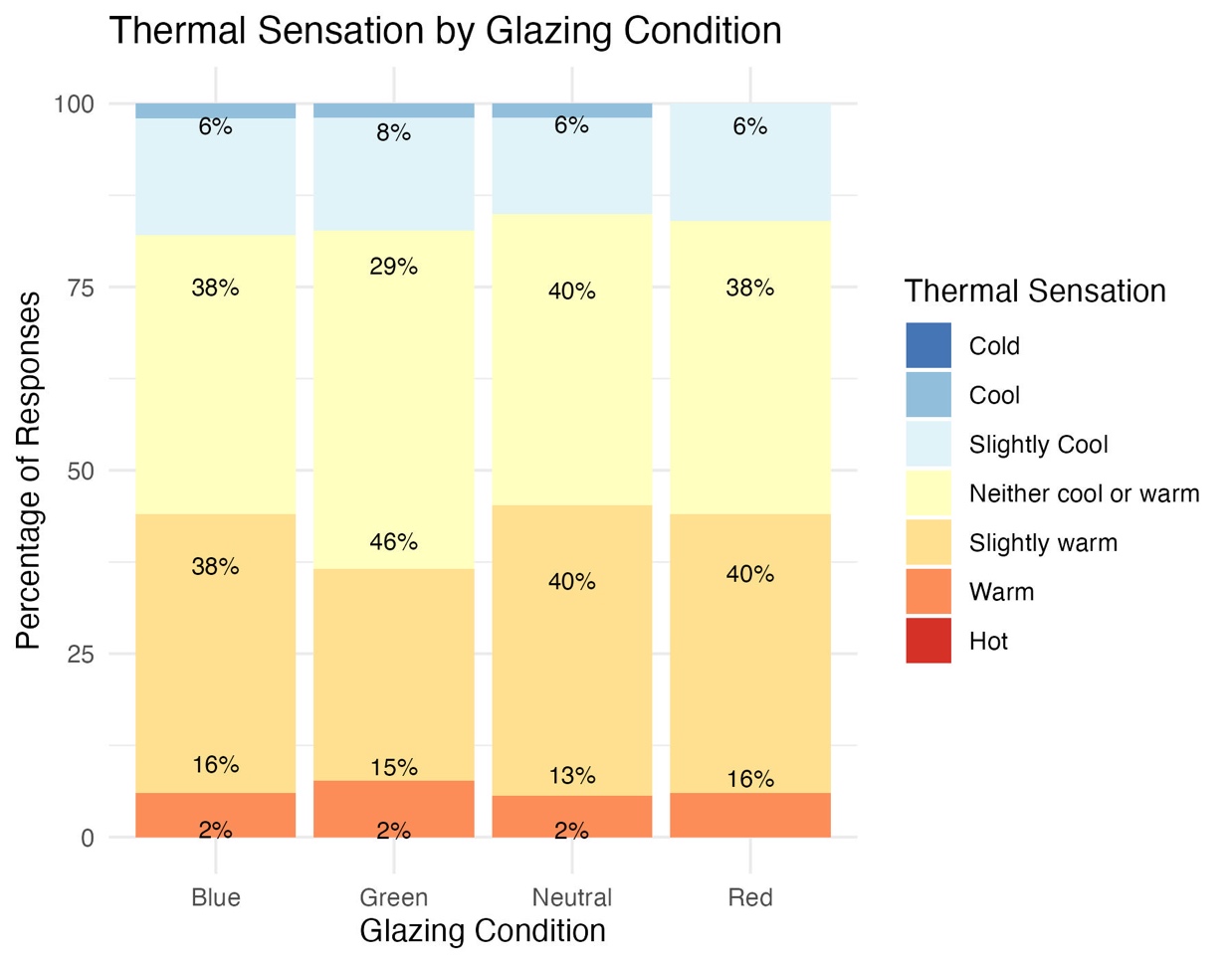


**Supplementary Figure S4:** Boxplots with marked mean and median demonstrating the average vertical gaze direction during each exposure period separated by the color and intensity of the experimental conditions (left); p-value of Welch’s t test on each pair of boxplots for the low and high transmittance conditions (right)


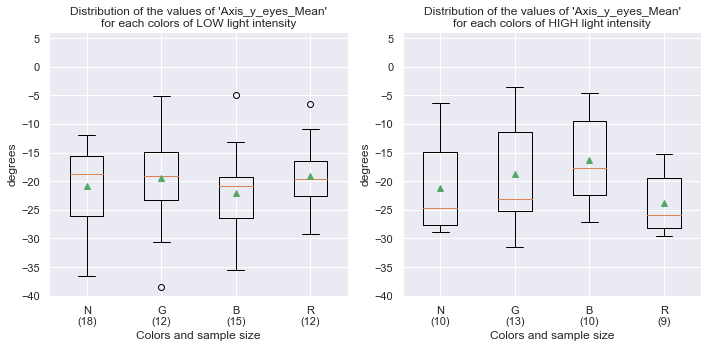

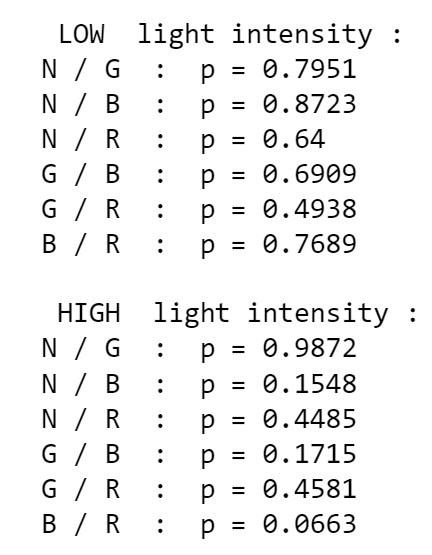


### **References**

1. Jain, S., Wienold, J., Lagier, M., Schueler, A. & Andersen, M. Perceived glare from the sun behind tinted glazing: Comparing blue vs. color-neutral tints. *Building and Environment* **234**, 110146 (2023).

2. Suzuki, Y., Minami, T., Laeng, B. & Nakauchi, S. Colorful glares: Effects of colors on brightness illusions measured with pupillometry. *Acta Psychologica* **198**, 102882 (2019).

3. Niedling, M. & Völker, S. Influence of a glare sources spectrum on discomfort glare – a physiological explanation for a psychological phenomenon. In *proceedings of the conference at the cie midterm meeting 2017 23 – 25 october 2017, jeju, republic of korea* 866–870 (International Commission on Illumination, CIE, Jeju Island, Republic of Korea, 2018). doi:10.25039/x44.2017.PO21.

4. Choi, S. & Ko, J. K. Contents development for office smart lights. In *proceedings of the conference at the cie midterm meeting 2017 23 – 25 october 2017, jeju, republic of korea* 1–9 (International Commission on Illumination, CIE, Jeju Island, Republic of Korea, 2018). doi:10.25039/x44.2017.IP01.

5. Yang, Y., Luo, R. M. & Huang, W. J. Assessing glare, Part 3: Glare sources having different colours. *Lighting Research & Technology* 1477153516676640 (2016) doi:10.1177/1477153516676640.

6. Chen, P.-L. *et al.* A portable inspection system to estimate direct glare of various LED modules. in (eds. Asundi, A. K. & Fu, Y.) 95241X (Singapore, Singapore, 2015). doi:10.1117/12.2189599.

7. Wei, M. *et al.* Field study of office worker responses to fluorescent lighting of different CCT and lumen output. *Journal of Environmental Psychology* **39**, 62–76 (2014).

8. Zhang, J. *et al.* P.30: Effect of the Correlated Color Temperature of Light on Overhead Glare in Offices. *SID Symposium Digest of Technical Papers* **44**, 1096–1098 (2013).

9. Sweater-Hickcox, K., Narendran, N., Bullough, J. D. & Freyssinier, J. P. Effect of different colored background lighting on LED discomfort glare perception. (2012).

10. Kimura-Minoda, T. & Ayama, M. Evaluation of Discomfort Glare from Color LEDs and Its Correlation with Individual Variations in Brightness Sensitivity. *Color research and application* **36**, 286–294 (2010).

11. Fekete, J., Sik-Lanyi, C. & Schanda, J. Spectral discomfort glare sensitivity investigations. *Ophthalmic and Physiological Optics* **29**, 1–6 (2009).

12. Bullough, J. D. Spectral sensitivity for extrafoveal discomfort glare. *Journal of Modern Optics* **56**, 1518–1522 (2009).

13. Sivak, M. *Blue Content of LED Headlamps and Discomfort Glare*. http://deepblue.lib.umich.edu/handle/2027.42/57444 (2005).

14. Bullough, J. D., Van Derlofske, J., Dee, P., Chen, J. & Akashi, Y. An investigation of headlamp glare: intensity, spectrum and size. (2004).

15. Flannagan, M. J. *Subjective and Objective Aspects of Headlamp Glare: Effects of Size and Spectral Power Distribution*. 19 (1999).

16. Berman, S. M., Bullimore, M. A., Bailey, I. L. & Jacobs, R. J. The Influence of Spectral Composition on Discomfort Glare for Large-Size Sources. (1995).

17. Flannagan, M. J., Sivak, M., Ensing, M. & Simmons, C. J. *Effect of Wavelength on Discomfort Glare from Monochromatic Sources.* http://deepblue.lib.umich.edu/handle/2027.42/64064 (1989).

18. CEN. Glass in building - Determination of luminous and solar characteristics of glazing; German version EN 410:2011. (2011) doi:https://dx.doi.org/10.31030/1747600.

19. R. Steiner, Oelhafen, P., Reber, G. & Romanyuk, A. Experimental determination of spectral and angular dependent optical properties of insulating glasses. in *CISBAT Proceedings* 441–446 (EPFL, Lausanne, Switzerland, 2005).
